# Effects of early, combined endurance and resistance training in mechanically ventilated, critically ill patients: A randomised controlled trial

**DOI:** 10.1371/journal.pone.0207428

**Published:** 2018-11-14

**Authors:** Sabrina Eggmann, Martin L. Verra, Gere Luder, Jukka Takala, Stephan M. Jakob

**Affiliations:** 1 Department of Physiotherapy, Inselspital, Bern University Hospital, Bern, Switzerland; 2 Department of Intensive Care Medicine, Inselspital, Bern University Hospital, University of Bern, Bern, Switzerland; University of Bern, SWITZERLAND

## Abstract

**Introduction:**

Neuromuscular weakness resulting in severe functional impairment is common in critical care survivors. This study aimed to evaluate effects of an early progressive rehabilitation intervention in mechanically ventilated adults at risk.

**Methods:**

This was a parallel, two-arm, assessor-blinded, randomised controlled trial with 6-months follow-up that was conducted in a mixed ICU of an academic centre in Switzerland. Previously independent, mechanically ventilated, critically ill adults with expected critical care stay ≥72 hours (n = 115) were randomised to a control group receiving standard physiotherapy including early mobilisation or to an experimental group with early endurance and resistance training combined with mobilisation. Primary endpoints were functional capacity (6-Minute Walk Distance) and functional independence (Functional Independence Measure) at hospital discharge. Secondary endpoints including muscle strength were assessed at critical care discharge. Safety was monitored closely by standard monitoring and predefined adverse events.

**Results:**

Physiotherapy started within 48 hours of critical care admission while 97% of participants were still ventilated and 68% on inotropes. Compared to the control group (n = 57), the experimental group (n = 58) received significantly more physiotherapy (sessions: 407 vs 377, p<0.001; time/session: 25min vs 18min, p<0.001) and had less days with sedation (p<0.001). Adverse events were rare (0.6%) and without consequences. There were no significant between-group differences in 6-Minute Walk Distance (experimental 123m (IQR 25–280) vs control 100m (IQR 0–300); p = 0.542) or functional independence (98 (IQR 66–119) vs 98 (IQR 18–115); p = 0.308). Likewise, no differences were found for the secondary outcomes, except a trend towards improved mental health in the experimental group after 6 months (84 (IQR 68–88) vs 70 (IQR 64–76); p = 0.023).

**Conclusions:**

Early endurance and resistance training in mechanically ventilated, intensive care patients does not improve functional capacity or independence at hospital discharge compared to early standard physiotherapy but may improve mental health 6-months after critical care discharge.

**Trial registration:**

German Clinical Trials Register (DRKS): DRKS00004347, registered on 10 September 2012.

## Introduction

Intensive care unit acquired weakness (ICUAW) is common in critical illness survivors. It increases health-care costs and impairs quality of life [1]. ICUAW mainly affects skeletal muscles (critical illness myopathy) and peripheral nerves (critical illness polyneuropathy) [2], but its causes are poorly understood [3]. Symptoms of ICUAW develop within the first week of critical illness [4]. Its risk factors include sepsis, multiple organ failure, mechanical ventilation (MV), immobilisation and hyperglycaemia [3]. ICUAW is associated with worse short- and long-term outcomes, including weaning failure [5], prolonged ICU and hospital stays [6], increased mortality [7], and poor functional status with persistent disability in activities of daily living [1, 8–10].

Early rehabilitation and mobilisation may help to reduce the functional disabilities of ICUAW patients. Some promising short-term outcomes have been reported [11, 12], but long-term benefits from randomised controlled trials are warranted [13–15]. Delayed intervention onset (≥1 week) [12, 13, 15], low ICUAW-risk in targeted population [11] or unspecific exercise delivery [11, 14] may have contributed to the inconclusive results. Despite these limitations in evidence, many ICUs, including ours, have adopted early mobilisation, also as a consequence of using less sedation. We aimed to investigate a specific rehabilitation intervention that could be implemented early in a highly ill population with a high ICUAW risk.

Resistance training can restore strength after bed-rest in older adults [16]. Combined endurance and resistance training (ERT) enhances exercise capacity and health-related quality of life in patients with chronic obstructive pulmonary disease [17] and prevents cardiovascular and skeletal muscle deconditioning during bedrest [18]. Whether early ERT is feasible, safe and beneficial in critically ill patients of whom many are still mechanically ventilated and treated with opioids or recovering from sedation is unclear. We hypothesised that early ERT combined with mobilisation in the ICU is feasible and might improve function at hospital discharge. This randomised controlled trial compared the effects and safety of early ERT combined with early mobilisation to standard care including early mobilisation in critically ill, mechanically ventilated adults for a period of up to 6-months after hospital discharge.

## Methods

### Design and setting

This single-centre, parallel, two-arm, assessor-blinded randomised controlled trial with 6-months follow-up was conducted in the tertiary, mixed ICU of the Department of Intensive Care Medicine at the Inselspital, Bern University Hospital, Switzerland. The trial was approved by the local Ethics Committee and prospectively registered in the German Clinical Trials Register (DRKS00004347) on September 10, 2012. It adhered to Good Clinical Practice Guidelines and the CONSORT statement (S1 Checklist). The study protocol has previously been published [19].

### Participants

Adults (≥18 years) expected to stay on MV for at least 72 hours and who had been independent before the onset of critical illness were eligible for participation. Independence was defined as living without assistance and qualitatively determined by the patient’s family or medical-health records. Exclusion criteria were previous muscle weakness, contraindications to cycling, enrolment in another intervention study, palliative care, admission diagnosis that excluded the possibility of walking at hospital discharge, and patients who did not understand German or French [19].

### Procedures

Patients were screened daily by a research nurse and a specialist physiotherapist who evaluated cycling eligibility. The expected MV duration was judged by the responsible physician. Subsequently and before study inclusion, an independent medical doctor confirmed eligibility criteria. Physicians and physiotherapists were unaware of the randomisation sequence. A deferred consent was obtained from the next of kin within 72 hours after randomisation and a written informed consent from each patient as early as possible. Unrestricted, computer-generated randomisation (https://www.random.org/lists/) was performed by a study nurse partly involved in screening eligible candidates. Participants were allocated at a 1:1 ratio to either group using sequentially numbered opaque sealed envelopes to ensure allocation concealment [20]. Coded data was managed with REDcap [21].

Blinding the responsible ICU staff was impossible, but the physiotherapy assessors were blinded for group allocation and distinct from the therapists providing the intervention. However, logistical reasons prevented therapist separation between the two treatment groups. Discharge decisions were made by the primary care team without the involvement of physiotherapists.

### Interventions

Both groups received standard ICU care with protocol-guided sedation, weaning [22] and nutrition [23]. In cases of ICU readmissions, treatment according to initial group allocation was continued.

#### Standard care (control group)

Patients in the control group received European standard physiotherapy [24] including early mobilisation, respiratory therapy and passive or active exercises. These were physiotherapy-initiated and individually tailored, but subject to a medical prescription. Treatments took place once daily on weekdays and on weekends if the responsible therapists deemed an interruption of therapy to be harmful to patient’s previous progress (e.g. intensive rehabilitation and weaning period or retained airway secretion in extubated patients).

#### Study intervention (experimental group)

In patients in the experimental group an early, progressive ERT programme combined with early mobilisation (S1 Fig) was initiated and each component implemented as intensive as possible and tolerated in individual patients. To this end, sedation was reduced if medically permitted prior to physiotherapy [19]. Physiotherapists were encouraged to split therapy into two or more sessions to prevent overexertion. Therapy visits occurred from Monday to Friday up to a maximum of three sessions per day and on weekends if deemed helpful to patient’s progress.

Endurance training was conducted with a motor-assisted bed-cycle (MOTOmed letto2, Reck-Technik, Betzenweiler, Germany) that allows passive, motor-assisted or active cycling in bed. Patients were comfortably positioned in a supine position with an individual head-of-bed elevation to permit optimal leg movement. Maximal training intensity for unresponsive patients was 20 minutes with a pedalling rate of 20 cycles/min [12]. During each session, patients were prompted verbally and with the MOTOmed ServoCycle feature to actively participate. When patients were able to cycle with motor-assistance for 20 minutes, assistance was gradually decreased. Only then was training duration raised to 30 minutes. Subsequently, resistance and then duration were increased with a defined maximum of 60 minutes with full resistance (S1 Table).

Resistance training included standardised exercises for both upper and lower limbs using weights or manually administered resistance from the therapist. The targeted training intensity was 8–12 repetitions with 2–5 sets (2min rest) on 50–70% of the estimated one-repetition maximum. Once the exercises were performed properly, participants were given an illustrated handout for further training with family members or nurses. In patients unable to perform resistance exercises because of lack of strength or comprehension, physiotherapists used passive movement or tactile facilitation to excite movement.

Early mobilisation was started with in-bed exercises and, if no medical contraindications were present, continued progressively from sitting on the bedside to sitting in a chair to standing and, finally, to walking. The overall aim was patients’ active participation in functional activities of daily living in order to promote early independence.

Usual physiotherapy was provided for patients after ICU discharge once to twice a day. Sessions commonly involved functional exercises, cycling, walking, strengthening and breathing techniques.

### Outcomes

The two primary outcomes were functional capacity (6-Minute Walking Distance (6MWD) [25]) and the ability to perform activities of daily living (Functional Independence Measure (FIM) [26]) at hospital discharge.

Secondary outcomes were FIM and muscle strength at ICU discharge. Muscle strength was assessed if patients could follow at least 3 out of 5 standardised questions [27]. Assessments included the Medical Research Council (MRC) sum-score for ICUAW diagnosis [2], handgrip strength using the JAMAR dynamometer (Sammons Preston Rolyan, Bolingbrook, IL, USA) [28] and quadriceps muscle strength measured with a handheld dynamometer (Microfet 2, Biometrics, Almere, Netherlands) [29]. Additionally, limitations in range-of-motion were recorded for shoulder flexion, elbow flexion and extension, fist closure, hip flexion, knee flexion and extension and foot dorsiflexion [30]. To assess functional mobility the Timed ‘Up & Go’ was performed at hospital discharge [31]. Further endpoints were time on MV, ICU and hospital length of stay along with achieved ICU mobility. Quality of life was determined with the Short Form 36 (SF-36) six months after hospital discharge. The SF-36 is a valid and reliable measurement to assess long-term physical and mental health in ICU survivors [32].

To judge training intensity and stability, physiological trends and individually-set limits instead of strict target numbers were used to account for each patient’s condition and variability. Safety was closely monitored using standard ICU monitoring and indirect calorimetry for 30min before, during and 15min after physiotherapy. Physiological data was recorded in a patient data management system that stored 2-minute median values to remove artefacts. Prospectively defined adverse events (AE) included new hemodynamically relevant arrhythmias or otherwise unstable hemodynamics, oxygenation desaturation under 85%, a fall or other injury and any accidental removal of a tube, catheter or similar device. Per definition, AEs occurred during or up to 15min after physiotherapy and persisted despite an intervention or therapy interruption. Other known complications of bedrest, e.g. atelectasis or pressure ulcers, were recorded along with any serious AE which included death, a life-threatening injury or any event that caused an extension of hospital stay.

### Statistical analysis

Sample size determination was based on the 6MWD to show a difference of 54m [33] and a mean walking distance of 301m (SD 81) [34]. A statistical power of 80% and an α-level of 0.05 required a sample size of totally 72 patients (36 per group). This was further adjusted by 28 patients, due to two primary outcomes that were expected to highly correlate. Lastly, the overall sample size was increased by 15% to 115 participants because of expected attrition, including mortality. It was decided a priori that refusals to participate (presumed consent not obtained within 72h of randomisation) were compensated for and were not to be included in the intention-to-treat analysis. The number of these additional participants was determined after the inclusion of 115 patients. As a consequence, 130 patients were randomised until the number of 115 participants with presumed consent was reached.

Analysis was by intention-to-treat and per protocol using SPSS (IBM SPSS Statistics Version Premium GradPack 24), and R (Version 3.4.4). In the per protocol analysis, patients who died were allocated the lowest possible outcome for both primary outcomes. The intention-to-treat analysis was conducted with multiple imputation using a linear regression model that imputed all missing values (including death). Sensitivity analyses for both primary outcomes were conducted to test the robustness of the main analysis. Secondary outcomes were analysed as measured. Between group comparisons for normally distributed data included Student’s t-tests and for non-normally distributed data Mann-Whitney-U tests. Dichotomous data was analysed with Fisher’s exact test, repetitive data with repeated measures analysis of variance (ANOVA) and correlations with Spearman correlation coefficients. Continuous data are presented as means with standard deviation (SD) or median with interquartile range (IQR) dependent upon their distribution. Categorical data are given as numbers with percentages. Treatment effects are reported with 95% Confidence intervals (95% CI) as mean differences (MD) for normally distributed data, as estimator for difference in location for non-normal data (Wilcoxon-Mann-Whitney test implemented in R package “coin”), and as incidence rate ratios (IRR) or risk differences (RD) for percentages. IRRs and RDs were calculated with R using Exact Poisson and Proportion test, respectively. Effect sizes were calculated with Cohen’s d for normally distributed data and with Cohen’s r for Wilcoxon-Mann-Whitney test statistic in case of non-normal data (using appropriate z value as in [35]). The level of significance was set at p < 0.05. A preliminary safety analysis has been conducted after the enrolment of 35 participants [36].

## Results

### Study participants

**Fig 1:**
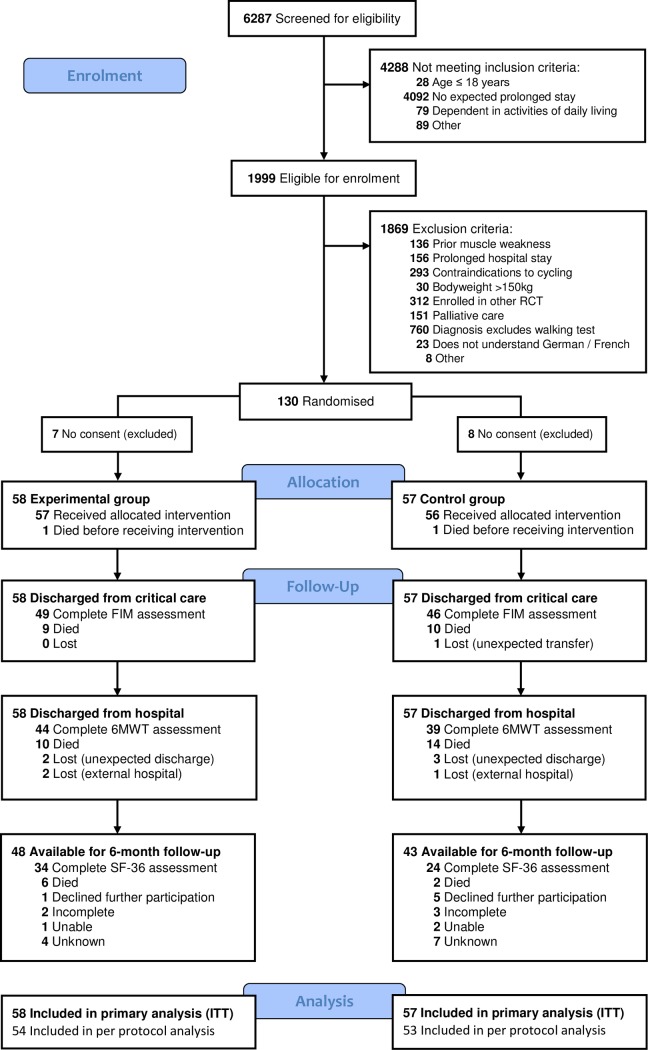
Study flow diagram (CONSORT).

From October 8, 2012 to April 5, 2016, we randomised 130 patients for whom 15 (12%) patient’s proxy declined initial study participation. These were excluded from all further analyses, as requested by the Ethics Committee. Consequently, 115 patients (experimental n = 58, control n = 57) were included in the intention-to-treat analysis. (Fig 1). Baseline demographics and clinical characteristics were similar between groups (Table 1).

**Table 1:** Baseline demographic and clinical characteristics.

	Experimental group n = 58	Control group n = 57
Age, years, mean (SD)	65±15	63±15
Sex, female, n (%)	22 (38%)	16 (28%)
Weight, kg, mean (SD)	82±18	80±16
BMI, kg/m^2^, mean (SD)	28±6	27±4
APACHE II score, mean (SD) ^a^	22±8	23±7
TISS-28 score, median (IQR)	37 (31–42)	37 (32–44)
TISS-76 score, median (IQR)	46 (39–56)	48 (41–56)
ICU days until study inclusion, mean (SD)	1.9±1.2	1.8±1.2
SOFA score, median (IQR) ^b^	8 (6–10)	8 (7–12)
**ICU diagnosis on ICU admission**		
Heart surgery	13 (11%)	8 (7%)
Neurology / neurosurgery	5 (4%)	4 (3%)
Other surgery	5 (4%)	9 (8%)
Gastroenterology	7 (6%)	7 (6%)
Trauma	1 (1%)	3 (3%)
Respiratory insufficiency	13 (11%)	12 (10%)
Hemodynamic insufficiency	13 (11%)	13 (11%)
Other	1 (1%)	1 (1%)
**Comorbidities on ICU admission** (n = 111: experimental n = 54, control n = 57)		
NYHA symptoms (stage 2 to 4)	22 (41%)	26 (46%)
Dyspnoea symptoms	15 (28%)	15 (26%)
Hematologic malignancy	0 (0%)	5 (9%)
Immunosuppression	4 (7%)	12 (21%)
Liver disease	9 (17%)	6 (11%)
Chronic dialysis	0 (0%)	0 (0%)
Restricted in activities of daily living ^c^	8 (15%)	5 (9%)
**Laboratory on ICU admission**		
CRP, mg/l, median (IQR), n = 106	95 (49–276)	76 (30–147)
Creatinine, μmol /l, median (IQR), n = 109	97 (70–168)	111 (78–156)
Bilirubin, μmol /l, median (IQR), n = 68	15 (8–54)	18 (8–32)
Thrombocytes, 10^9^/L, median (IQR), n = 113	117 (102–216)	108 (67–148)
Leucocytes, 10^9^/L, median (IQR), n = 133	11 (8–16)	11 (6–16)
Haemoglobin, g/L, median (IQR), n = 115	109 (97–119)	102 (85–115)

^a^ at ICU admission

^b^ at study inclusion

^c^ inability to walk for longer distances or to participate in social life

Data are presented as median (IQR), mean (SD) or n (%)

**Abbreviations:** NYHA = New York Heart Association, BMI = Body Mass Index, APACHE = Acute Physiology and Chronic Health Evaluation, TISS = Therapeutic Intervention Scoring System, SOFA = Sequential Organ Failure Assessment, CRP = C-reactive protein

### Primary outcomes

No significant differences were found between groups in the intention-to-treat and the per protocol analysis (Table 2). There was a high positive correlation (p<0.001, r = 0.833) between the two primary outcomes. Results were robust in sensitivity analyses (S2 Table).

**Table 2:** Results for the two primary outcomes per protocol and intention-to-treat analysis.

	Experimental group n = 58		Control group n = 57		p value	Effect size	Group difference (95% CI)
	n	value	n	value			
**Per Protocol analysis**							
6 Minute Walking Distance (m)	54	123 (IQR 25–280)	53	100 (IQR 0–300)	p = 0.542	0.006	0.00 (-35.00 to 65.00)
FIM (18–126)	54	98 (IQR 66–119)	52	98 (IQR 18–115)	p = 0.308	0.010	3.00 (-2.00 to 14.00)
**Intention-to-treat analysis**^a^							
6 Minute Walking Distance (m)	58	223±133	57	246±167	p = 0.448	-0.151	-22.75 (- 81.66 to 36.16)
FIM (18–126)	58	101±22	57	99±24	p = 0.659	0.085	1.98 (-6.85 to 10.82)

^a^ Multiple imputation from a linear regression model based on age, gender, BMI, weight, APACHE II score, TISS-28, Tiss-76, SOFA score, ICU days until randomisation, length of stay in ICU and hospital, duration of mechanical ventilation, FIM score and MRC sum-score at ICU discharge. All missing values (including death before hospital discharge) were imputed (6MWD: n = 33, FIM: n = 32).

### Secondary outcomes

There were no significant differences between groups at ICU and hospital discharge (Table 3). The incidence of ICUAW at ICU discharge, determined by MRC sum-score in cooperative patients, was 58% (23/40) in the experimental and 61% (26/43) in the control group (n = 83; p = 0.826). Limitations in range-of-motion were observed in 22 (38%) patients of the experimental and in 16 (28%) of the control group (n = 115; p = 0.323) with a median of 2 (IQR 0–3) affected joints per patient. No joint was particularly vulnerable for contracture. There were no significant differences for ICU (p = 0.694) and hospital discharge destinations (p = 0.555) between groups. The most frequent discharge destinations for survivors were rehabilitation (40%; experimental n = 25, control n = 21), local hospital (26.1%; experimental n = 17, control n = 13) and home (15.7%; experimental n = 7, control n = 11). Except for mental health (p = 0.023), there were no significant differences in quality of life at 6-months after hospital discharge (Table 3). Because of substantial missing data for the SF-36, characteristics for patients with and without the 6-month follow-up were compared. There were no significant differences between patients that completed the SF-36 versus patients that did not return the questionnaire (S3 Table).

**Table 3:** Results of secondary outcomes.

	Experimental group n = 58		Control group n = 57		p value	Effect size	Group difference (95% CI)
	n	value	n	value			
**Hospital discharge**							
Timed “Up & Go” Test (s)	36	19.5 (IQR 11.5–25.0)	28	16.0 (IQR 10.3–29.0)	p = 0.538	0.010	1.70 (-3.70 to 7.00)
Time on mechanical ventilation (days)	58	5.4 (IQR 3.3–12.9)	57	5.0 (IQR 3.6–11.9)	p = 0.830	-0.002	-0.17 (-1.65 to 1.49)
Length of stay ICU (days)	58	6.1 (IQR 4.0–12.3)	57	6.6 (IQR 4.6–14.7)	p = 0.568	-0.005	-0.44 (-2.27 to 1.20)
Length of stay hospital (days)	58	25.9 (IQR 14.3–37.2)	57	22.0 (IQR 15.0–39.2)	p = 0.723	0.003	1.31 (-4.85 to 7.92)
**ICU discharge**							
FIM (18–126)	58	28.5 (IQR 21.0–42.0)	56	28.5 (IQR 19.5–41.5)	p = 0.791	0.002	0.00 (-4.00 to 5.00)
MRC sum-score (0–60)	40	42.4±13.1	43	44.4±11.7	p = 0.461	-0.161	MD -2.02 (-7.45 to 3.41)
Handgrip strength (JAMAR) (kg)	30	20.5±12.6	40	19.6±13.6	p = 0.780	0.069	MD 0.90 (-5.47 to 7.25)
Quadriceps strength (HDD) (kg)	28	7.7±4.0	33	8.0±3.9	p = 0.771	-0.076	MD -0.30 (-2.32 to 1.73)
**6-month follow-up: SF-36**							
Physical functioning (0–100)	36	75.0 (IQR 45.0–85.0)	27	75.0 (IQR 50.0–85.0)	p = 0.676	-0.007	-1.11 (-15.00 to 10.00)
Role physical (0–100)	36	25.0 (IQR 0.0–62.5)	25	33.3 (IQR 25.0–50.0)	p = 0.443	-0.013	0.00 (-25.00 to 0.00)
Bodily pain (0–100)	36	80.0 (IQR 51.0–100.0)	27	74 (IQR 41.0–100.0)	p = 0.530	0.010	0.00 (-9.00 to 20.00)
General health (0–100)	36	57.9±18.4	25	61.1±19.0	p = 0.513	-0.162	MD -3.20 (-12.91 to 6.52)
Vitality (0–100)	36	55.4±19.4	26	48.3±20.1	p = 0.167	0.359	MD 7.10 (-3.06 to 17.26)
Social functioning (0–100)	36	75.0 (IQR 65.3–100.0)	26	68.8 (IQR 50.0–100.0)	p = 0.458	0.012	0.00 (0.00 to 25.00)
Role emotional (0–100)	35	66.7 (IQR 33.3–100.0)	26	50.0 (IQR 0.0–100.0)	p = 0.870	0.003	0.00 (0.00 to 33.33)
Mental health (0–100)	35	84.0 (IQR 68.0–88.0)	26	70.0 (IQR 64.0–76.0)	p = 0.023	0.037	12.00 (0.00 to 16.00)
Physical health (sum-score)	34	40.8±11.1	24	42.7±10.4	p = 0.520	-0.190	MD-1.87 (-7.65 to 3.91)
Mental health (sum-score)	34	49.4±10.3	24	45.2±11.4	p = 0.147	0.381	MD 4.23 (-1.53 to 9.99)

Data are presented as median (IQR), mean (SD) or n (%)

**Abbreviations:** FIM = Functional Independence Measure (worst score 18: dependent, best score 126: independent), HHD = handheld dynamometer, LOS = length of stay, SF-36 = Short Form 36, version 2 (worst score: 0, best score: 100, sum-score: T-values where the population mean is 50 and the SD is 10; based on US-population 1990), MD = Mean Difference

### Safety

Safety details are given in Table 4. Overall, 25 (3%) of all physiotherapy sessions had to be discontinued (between-groups difference p = 0.839). The most frequent reasons for therapy discontinuation were exceedance of individually set limits (n = 8, 32%), lack of patient cooperation (n = 5, 20%) and fatigue (n = 3, 12%). There were 4 (0.6%) AEs, 1 (0.2%) in the experimental group due to an oxygen desaturation while cycling and 3 (0.8%) in the control group during mobilisation (one oxygen desaturation, two unstable haemodynamics). All AEs resolved after therapy discontinuation and did not have further consequences. The incidence of ICU complications did not differ between the two groups. ICU, hospital- and 6-month mortality rates were similar between groups over the study period.

**Table 4:** Safety details for the control and experimental groups.

	Experimental group (n = 58, 478 study days)	Control group (n = 57, 558 study days)	p value	Group difference (95% CI)
**Safety**				
PT discontinuation (% of total physiotherapy sessions)	12 **(3%)**	13 **(3%)**	p = 0.842	IRR 0.86 (0.36 to 2.03)
Adverse event (% per sessions)	1 **(0.2%)**	3 **(0.8%)**	p = 0.357	IRR 0.31 (0.01 to 3.85)
**Mortality**				
ICU mortality	9 **(16%)**	10 **(18%)**	p = 0.770	RD -2% (-16 to 12%)
In-hospital mortality	10 **(17%)**	14 **(25%)**	p = 0.334	RD -8% (-22 to 7%)
Mortality after 180 days	16 **(28%)**	16 **(28%)**	p = 0.954	RD 0% (-17% to 16%)
**ICU complications**^a^				
Delirium-free ICU days	256 **(61%)**	300 **(64%)**	p = 0.524	IRR 0.95 (0.80 to 1.12)
Atelectasis-free ICU days	334 **(93%)**	402 **(91%)**	p = 0.739	IRR 1.03 (0.88 to 1.19)
Pneumonia-free ICU days	293 **(94%)**	355 **(92%)**	p = 0.782	IRR 1.02 (0.87 to 1.20)
Thrombosis-free ICU days	415 **(96%)**	473 **(94%)**	p = 0.788	IRR 1.02 (0.90 to 1.17)
Decubiti-free ICU days	411 **(96%)**	493 **(95%)**	p = 0.867	IRR 1.01 (0.89 to 1.16)
Contracture-free ICU days	448 **(96%)**	535 **(96%)**	p>0.999	IRR 1.00 (0.88 to 1.13)

^a^ Assessed by responsible physician without any prespecified criteria, assessable ICU days only (discretion of physician) (delirium: experimental n = 421, control n = 467, atelectasis: n = 258, n = 442, pneumonia: n = 313, n = 388, thrombosis: n = 431, n = 501, decubiti: n = 427, n = 518, contracture: n = 468, n = 558)

Data are presented as n (%)

**Definitions:** Study days = number of days from study enrolment to ICU discharge including readmissions and weekends where only limited therapy service was available, session = one single physiotherapy treatment

**Abbreviations:** IRR = Incidence rate ratio, RD = Risk difference, PT = Physiotherapy, ICU = Intensive care unit

Patients on MV (S1 File) had a significant effect over time (30min before, during and 15min after physiotherapy) for mean arterial pressure (MAP; p<0.001, effect size η2 = 0.192), heartrate (HR; p<0.001, η2 = 0.161) and oxygen consumption (VO_2_; p<0.001, η2 = 0.224), but not for peripheral oxygen saturation (SpO_2_; p = 0.396, η2 = 0.017; repeated measures ANOVA). Post-hoc paired t-test with adjusted levels of significance (p<0.017) found increases from before to during physiotherapy for MAP (mean difference (95% CI): -1.83mmHg (-2.61 to -1.05), p<0.001), HR (-1.21beats/min (-1.76 to -0.65), p<0.001) and for VO_2_ (-12.22ml/min (-16.75 to -7.69), p<0.001). Additionally, there was an increase in HR from before to after physiotherapy (-0.83beats/min (-1.47 to -0.18), p = 0.012) and a decrease in VO_2_ from during to after physiotherapy (6.27ml/min (1.45 to 11.08), p = 0.011). Likewise, predominantly spontaneously breathing patients (S1 File) had a significant effect over time for MAP (p = 0.046, η2 = 0.073) and HR (p = 0.004, η2 = 0.123), but not for SpO_2_ (p = 0.083, η2 = 0.059). Post-hoc paired t-test with adjusted levels of significance (p<0.017) showed an increase from before to during physiotherapy for HR (-1.85 beats/min (-3.16 to -0.53), p = 0.006). There were no significant differences between groups for any of the vital parameters.

### Study interventions

The experimental group trained on 73% of study days (349 daily physiotherapy visits, including 407 individual sessions), whereas the control group received therapy on 65% of study days (365 visits, 377 sessions; Table 5). The main reasons for missed physiotherapy visits were weekends (188, 58%), day of ICU discharge (42, 13%), medical procedures (33, 10%) or cardiorespiratory instability (19, 6%). Patients in the experimental group had a median of 3.5 sessions (minimum 0, maximum 66) and patients in the control group of 3 sessions (minimum 0, maximum 39) (S2 File). Treatment session duration was slightly longer in the experimental group (25min (IQR 19–27) vs control 18min (IQR 14–21); p<0.001). This was recorded from the beginning to the end of actual therapy delivery and did not include preparation time, cleaning materials and documentation. On average, physiotherapy started within 48 hours (n = 113; median 1.98 days (IQR 1.41–2.86); experimental 47h (IQR32-68); control 52h (IQR 36–70); p = 0.468) after ICU admission and within 4 hours after randomisation. At this time 97% of participants were still ventilated (experimental 57(100%), control 52(93%); p = 0.057), 97% sedated (experimental 55(97%), control 55(98%), p<0.999), 68% on vasoactive support (experimental 42(74%), control 35(63%); p = 0.230), 19% paralysed (experimental 9(16%), control 12(21%); p = 0.477) and 97% received opioids (experimental 55(97%), control 54(96%); p<0.999). Patients in the experimental group were less likely to receive sedation (incidence risk ratio (IRR) 1.66 (1.26 to 2.18, p = 0.0002) and steroids (IRR 1.18 (1.03 to 1.35), p = 0.016), but more likely to receive vasopressors (IRR 0.77 (0.64 to 0.92), p = 0.005) compared to the control group.

**Table 5:** Treatment details for the control and experimental groups.

	Experimental group (n = 58, 478 study days)	Control group (n = 57, 558 study days)	p value	Group difference (95% CI)
**Physiotherapy (PT)**				
Total PT visits (% per study day)	349 **(73%)**	365 **(65%)**	p = 0.143	IRR 1.12 (0.96 to 1.30)
Total PT sessions (% per study day)	407 **(85%)**	377 **(68%)**	p<0.001	IRR 1.26 (1.09 to 1.45)
Two or more sessions per day (% of total PT sessions)	60 **(15%)**	15 **(4%)**	p<0.001	IRR 3.71 (2.08 to 7.03)
Treatment duration per session (min) ^a^	25.0 (IQR 19.5–27.0)	18.0 (IQR 14.0–21.0)	p<0.001	6.00 (4.00 to 8.00)
Time from enrolment to first therapy (days) ^b^	0.16 (IQR 0.07–0.20)	0.16 (IQR 0.07–0.48)	p = 0.387	-0.02 (-0.07 to 0.03)
Time from ICU admission to first therapy (days) ^b^	1.95 (IQR 1.41–2.80)	2.15 (IQR 1.53–2.89)	p = 0.470	-0.14 (-0.61 to 0.24)
**ICU treatment**				
Length of ICU stay at original hospital (days)	5.9 (IQR 4.0–10.9)	6.1 (IQR 4.6–12.7)	p = 0.595	-0.37 (-1.90 to 1.13)
Study days at original hospital	5.0 (IQR 3.0–10.0)	5.0 (IQR 4.0–12.0)	p = 0.696	0.00 (-2.00 to 1.00)
Tracheostomies	15 **(26%)**	15 **(26%)**	p = 0.956	RD 0% (-17% to 16%)
ICU re-admissions	8 **(14%)**	11 **(19%)**	p = 0.427	RD -5% (-19 to 8%)
Daily energy intake (kcal) over whole ICU stay ^c^	1225±833	1152±867	p = 0.139	MD 73.06 (-23.74 to 169.86)
**ICU medication**				
Study days without sedation	132 **(28%)**	93 **(17%)**	p<0.001	IRR 1.66 (1.26 to 2.18)
Study days without opiates	41 **(9%)**	56 **(10%)**	p = 0.477	IRR 0.85 (0.56 to 1.30)
Study days without insulin infusions	289 **(61%)**	333 **(60%)**	p = 0.872	IRR 1.01 (0.86 to 1.19)
Study days without NMBAs	407 **(85%)**	468 **(84%)**	p = 0.839	IRR 1.02 (0.89 to 1.16)
Study days without steroids	427 **(89%)**	422 **(76%)**	p = 0.016	IRR 1.18 (1.03 to 1.35)
Study days without inotropes and vasopressors	191 **(40%)**	291 **(52%)**	p = 0.005	IRR 0.77 (0.64 to 0.92)

^a^ Median of all patients’ median session duration

^b^ n = 57 for experimental and n = 56 for control group due to two participants death before receiving the allocated intervention

^c^ Excluding oral food intake

Data are presented as median (IQR), mean (SD) or n (%)

**Definitions:** Visits = days with at least one physiotherapy treatment, study days = number of days from study enrolment to ICU discharge including readmissions and weekends where only limited therapy service was available, session = one single physiotherapy treatment

**Abbreviations:** PT = Physiotherapy, ICU = Intensive care unit, NMBAs = Neuromuscular blocking agents, IRR = Incidence rate ratio, RD = Risk difference, MD = Mean Difference

**Fig 2:**
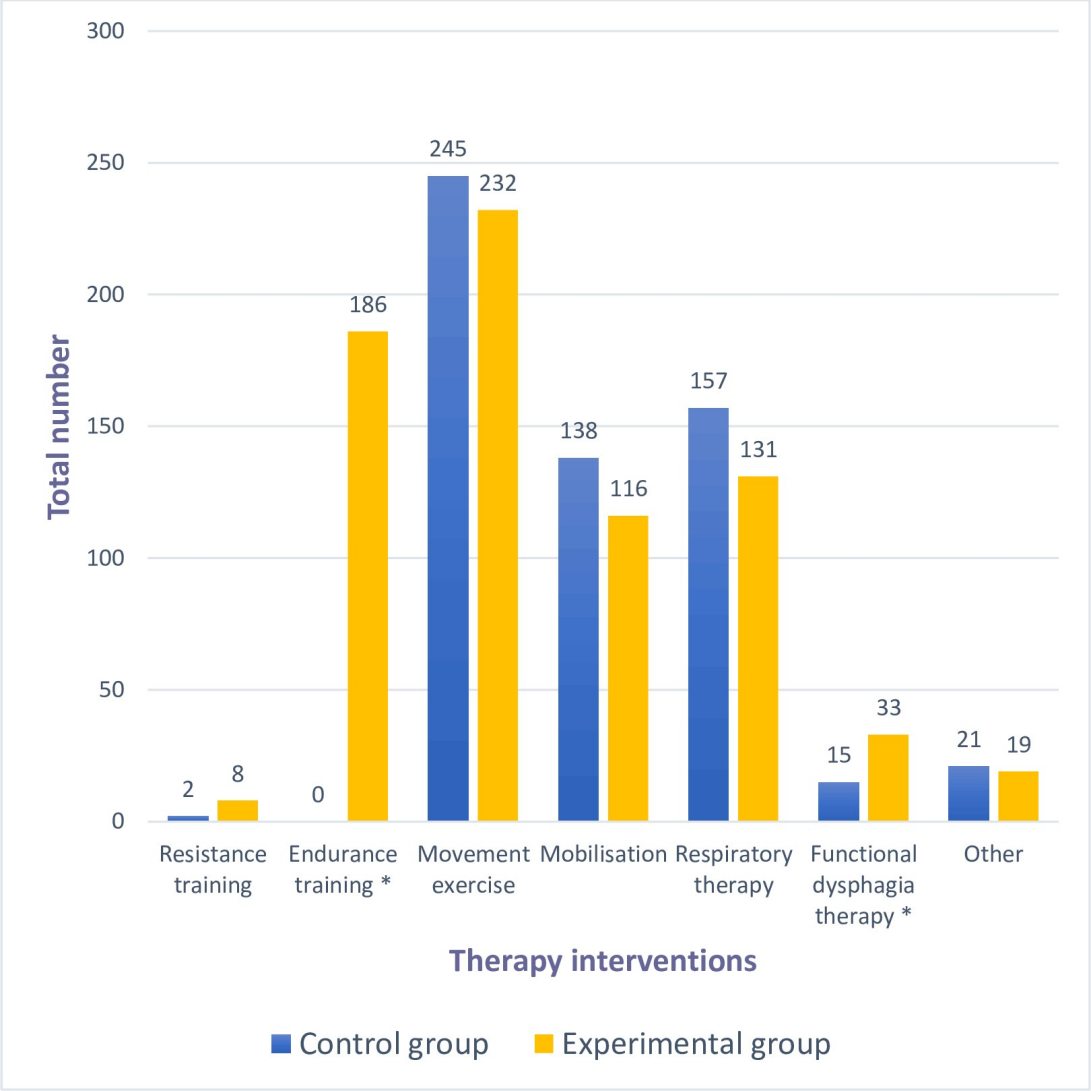
Overview of each physiotherapy intervention for the control and experimental groups. * p<0.003 More than one therapy intervention per session (= single physiotherapy treatment) possible, total sessions: 407 (85% of study days) experimental and 377 (68%) control group.

The number of each therapy intervention per group is outlined in Fig 2. Most patients were unable to perform the planned resistance training (sessions experimental 8, control 2; p = 0.052) with the exception of some long-stayers (S4 Table). Type of cycling included passive (141, 76%), mixed (28, 15%) and active training modes (17, 9%). Mean cycling distance was 1183 m (±721) during an average of 15min (±5), a pedalling rate of 20r/min (±7) and a resistance level of 0.2 (±0.8). Physiotherapy-initiated and overall mobilisations differed between groups; the experimental group had more edge-of-bed mobilisations, while the control group stood more (S2 File). However, achievement of milestones from time of randomisation to first edge-of-bed were not different (experimental 4d (IQR 2–7), control 5d (IQR 2–7); p = 0.451) (S2 File).

## Discussion

This randomised controlled trial did not find an advantage of early ERT combined with mobilisation over standard early mobilisation and rehabilitation. While participants in the experimental group received more therapy and had less days with sedation, control group participants had more out-of-bed mobilisations. Functional capacity and independence did not differ between these two different exercise regimes at hospital discharge. No differences were found in the secondary outcomes, except for a trend towards improved mental health at 6-months after hospital discharge. AEs were rare and a stepwise, individually-supervised training approach starting within 48 hours of ICU admission appears safe. ERT interventions are feasible and highly beneficial for functional improvement in chronic obstructive pulmonary disease [17] and we hypothesised that they may be equally feasible and effective in the critically ill. However, the transfer of these interventions may not be as straightforward as anticipated. Pulmonary rehabilitation is usually delivered over 8 to 12 weeks [17], while our exercise exposure was limited to the time spent in ICU, when patients were recovering from critical illness, mostly still on mechanical ventilation, receiving opioids and sedatives or at least recovering from previously received sedatives. Since the patients in our study spent the greater part of their hospital-stay on wards or step-down units (experimental 77%, control 70%) and the main reason for missed therapy visits were weekends (58%), extended protocol-exposure with more resistance training might have enhanced potential group differences. The median ICU-stay of 6 days seemed to have been too short to properly introduce resistance training, which was defined as using weight or resistance. However, assistive exercise (experimental group: 25% of movement exercises) in the very weak patient may affect strength as well. Wright et al [37] reported a higher but still modest percentage of sessions (33%) with this type of strengthening. In their study, similar to ours, sedation and patient fatigue prevented implementation or targeted intensity. Weakness (58% with ICUAW in our study) and ongoing effects of sedative agents despite significant reduction in their use in this group may both have contributed.

The main difference between our study and previous ones reporting benefits from early mobilisation is that the control group was also mobilised early, as per our ICU standard. We started the intervention very early in both groups, while patients were still mechanically ventilated and on vasoactive support, whereas most [12, 13, 15, 37, 38], but not all other intervention groups [11, 39] started substantially later. Nevertheless, our control group received physiotherapy as early as the intervention group in the study of Schweickert et al [11], and more often than treatment groups in other trials [37, 38]. In contrast to our study, Schaller et al [39] found that early mobilisation shortened ICU length of stay and improved functional mobility in surgical ICU patients at hospital discharge. Contribution of treatment of control groups to the different results cannot be evaluated due to insufficient details of care received in Schaller’s study. Yet the case mix appears very different: our patients were sicker (mean APACHE II 23 vs 16 in [39]) and had less surgical patients. It is therefore conceivable that the early and intensive physiotherapy in the control group of our study explains the lack of effect of additional endurance training. Early beginning of physiotherapy seems crucial due to early onset of ICUAW [4]. Accordingly, Schweickert et al [11] combined early mobilisation (within 1.7d of intubation) with daily sedation-interruption leading to reduced delirium and to significant functional improvement at hospital discharge. Delirium was equally prevalent in our groups, though the incidence was comparable to the intervention group of [11] (39 vs 33%). However, despite the early start, patients in our study achieved the specific mobilisation milestones substantially later [11]. This may also be related to the case mix–our patients were substantially older and sicker. These patients might not always be suitable for early mobilisation [40], but could profit from appropriate exercise interventions over the whole ICU stay [12]. However, recent evidence also highlights the impact of pre-ICU health [41, 42] and post-extubation dysphagia [43] on patients’ functional recovery. Our heterogenous sample might therefore conceal responsive subgroups. More research is needed whether sicker patients need longer rehabilitation exposure [44] and for which subgroups recovery will be achievable.

The conducted early endurance training seemed to have unfavourable effects on mobilisation levels leading to less out-of-bed mobilisations in this group. This self-funded, pragmatic study did not have additional resources for therapy-delivery. Physiotherapists may therefore have used available resources for cycling instead of early mobilisation. Although more out-of-bed mobilisations did not positively impact function of the control group either. The large AVERT trial even found that early, high-dose out-of-bed mobilisation after stroke enlarges the probability for disability when compared to low-dose, early mobilisation [45]. Dose-response analyses found the greatest benefit for short, frequent mobilisations [46]. This would favour the higher frequency of physiotherapy sessions (11%) in the intervention group, although this percentage might have been insufficient to reach clinical significance. Similarly, the stepwise approach of our study might have been too ambitious for the allocated therapy time (7min extra) and cycling insufficiently dosed (15min versus 30min reported by [47, 48]) to induce musculoskeletal adaptations. However, the small but significant changes in hemodynamic or oxygen-transport variables appeared to have been sufficient to stimulate patients to meet the metabolic demands of exercise by adequate cardiorespiratory adaptations. In contrast, neither passive cycling in deeply sedated patients [49] nor passive chair transfer [50] increased metabolic costs.

Our results are in line with recent studies [13, 15, 37, 38, 51] that failed to show a benefit of early rehabilitation. Heterogenous populations, difficulties in group separation and exercise dose may all have contributed. The discrepancy between planned and actual therapy delivery in these trials implies that the critically ill’s ability to participate in functional exercises may be overestimated. More attention should be paid to patient fatigue and on whom to target, as very early muscle preservation might not be feasible in a seriously ill cohort with active catabolic pathways. Resistance training may therefore be more feasible during recovery.

This study had several limitations. First, blinding of participants and physiotherapists was impossible. Second, loss of follow-up at 6 months was substantial and unequally distributed between groups (41% versus 58%) but without any differences in responders and non-responders. Nevertheless, results from the SF-36 are not adequately powered, possibly attributable to type I error and need to be interpreted with caution. Third, we targeted patients at risk of ICUAW who were previously independent. Therefore, data from this trial might not be applicable to patients with previous functional dependency. Fourth, the observed difference of 23m in the 6MWD may be of clinical significance [52], however, to test a difference of 25m (SD 150) would require at least 1128 participants. Similarly, the two primary outcomes should be adjusted for multiplicity to avoid type I error.

This study also has several strengths: patients were included very early after ICU admission and received physiotherapy within 48 hours. Physiotherapy interventions were pragmatic and reflect real-world resources. The novel use of individually set limits instead of strict target numbers allows for more rehabilitation flexibility and appears to be a safe approach with a low incidence of AEs (0.6%). Nevertheless, physiotherapists were experienced in ICU rehabilitation, which is shown in the exceptionally high control group participation. With a total of 1368 edge-of-bed and 1292 out-of-bed mobilisations on 1130 ICU days this trial further underlines the importance of ICU rehabilitation as a team effort. Additionally, the blinded outcome measurements were patient-centred, valid, reliable and relevant to the devastating consequences of ICUAW.

In the light of these data future studies should assure sufficiently large sample sizes, aim to increase exercise-exposure and ensure a targeted, frequent exercise delivery to the right patient. The effect of early exercise on mental health needs further evaluation, considering that one in two survivors might experience psychiatric symptoms [53].

## Conclusions

We did not find functional benefits of early exercise training over an active usual care rehabilitation in mechanically ventilated, critically ill adults. Successively-adapted, early exercise training seemed to be safe. Early endurance training might improve long-term mental health, but the most effective rehabilitation intervention to excite neuromuscular adaptations still needs to be determined.

## Supporting information

S1 CONSORT checklist (DOCX)

S1 Fig Intervention protocol for the experimental group [19]. (PDF)

S1 Table Intervention details for the experimental group. Details are based on CONSORT extension for non-pharmacological interventions and the TIDieR Checklist.
(PDF)

S2 Table Sensitivity analyses for the primary outcomes. (DOCX)

S3 Table Patient characteristics for ‘with versus without SF-36’ (mental health component). (PDF)

S4 Table Patient characteristics for ‘with versus without resistance training’. (PDF)

S1 File Physiological parameters while on mechanical ventilation (S2 Fig) and predominantly spontaneously breathing (S3 Fig) patients for before, during and after physiotherapy. (PDF)

S2 File Detailed information on performed study interventions: sessions, mobilisations and achieved mobility-milestones (S4 to S7 Fig, S5 Table). (PDF)

S3 File Original study protocol and English translation. This protocol has been approved by the local ethics committee prior to first patient recruitment.
(PDF)

S4 File Original, de-identified primary and secondary outcome variables. (XLSX)
