# Supplementary figures and images for: Effects of early, combined endurance and resistance training in mechanically ventilated, critically ill patients: A randomised controlled trial
Source: PLoS One. 2018 Nov 14;13(11):e0207428. doi: 10.1371/journal.pone.0207428 (PMC6235392; doi:10.1371/journal.pone.0207428)

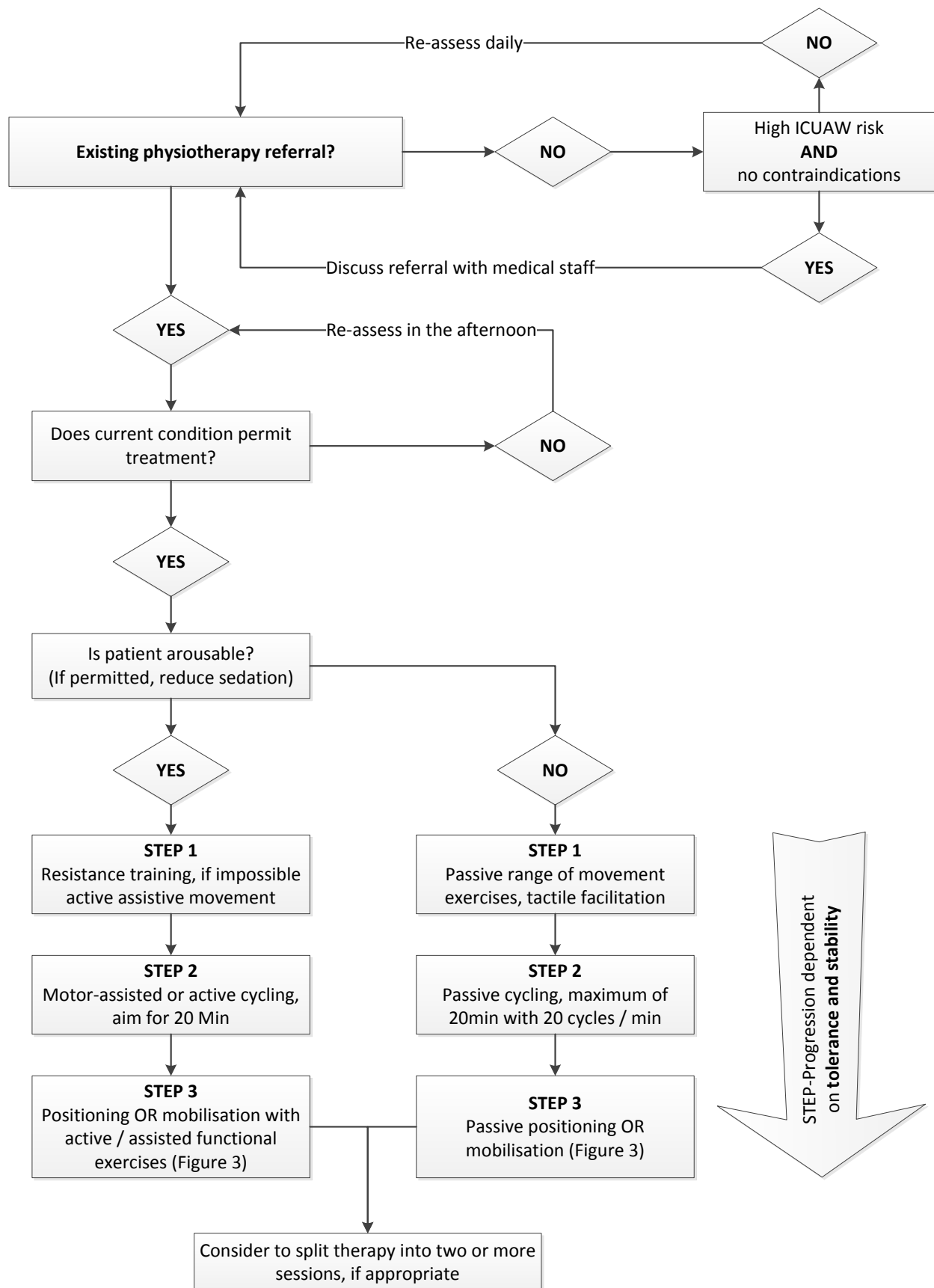

Supplement: S1 Fig — (PDF) [file pone.0207428.s002.pdf]
