# Supplementary material for: Effects of early, combined endurance and resistance training in mechanically ventilated, critically ill patients: A randomised controlled trial
Source: PLoS One. 2018 Nov 14;13(11):e0207428. doi: 10.1371/journal.pone.0207428 (PMC6235392; doi:10.1371/journal.pone.0207428)
Supplement: S1 Table — Details are based on CONSORT extension for non-pharmacological interventions and the TIDieR Checklist. (PDF) [file pone.0207428.s003.pdf]

S1 Table. Intervention details for the experimental group.

	Movement exercise	Resistance training	Endurance training
**Type**	Passive or assistive range of movement with tactile facilitation to excite movement	Three standardized exercises for upper and lower limbs: elbow flexion, arm push, external shoulder rotation, foot dorsiflexion, bridging, leg raise	Motor-assisted bed-cycle ergometer
**Time**	STEP 1 (Fig. 1) after study inclusion	Less than 25% support during assistive movement exercises	STEP 2 (Fig. 1), started if STEP 1 was tolerated
**Frequency**	Once daily on weekdays	Upper limbs on Monday, Wednesday, Friday. Lower limbs on Tuesday and Thursday.	Once daily on weekdays
**Intensity**	3-10 repetitions, 1-2 set, each joint, all directions	8-12 repetitions, 2-5 sets with 2min rest, 50-70% of estimated one-repetition maximum (weights starting from 450g or manual resistance)	If passive: maximum of 20min, 20 cycles/min<br>If active: maximum of 60min, level 6, target BORG level 11-13
**Who**	Certified physiotherapist	Certified physiotherapist	Certified physiotherapist (training was supervised)
**Tailoring**	If partial movement possible, assist to full range of motion	Individual exercises prescribed by responsible therapist	Achieve active partaking before adjusting assistance [a], increase time before resistance [b]
**Protocol breach**	Did not receive any exercise or resistance training	If impossible, conduct movement exercise	Did not cycle
**Adherence**	Number of sessions	Number of sessions	Number of sessions

[a] After 20min of active-assistive cycling decrease assistance (each session) to level 2

[b] Increase time to 30min, then increase resistance every second day to level 6, then increase time
