# Supplementary material for: Effects of early, combined endurance and resistance training in mechanically ventilated, critically ill patients: A randomised controlled trial
Source: PLoS One. 2018 Nov 14;13(11):e0207428. doi: 10.1371/journal.pone.0207428 (PMC6235392; doi:10.1371/journal.pone.0207428)
Supplement: S2 Table — (DOCX) [file pone.0207428.s004.docx]

**S2 Table. Sensitivity analyses for the primary outcomes (n=115).**

| **Variable** | **p value** | **Mean difference**  **(95% CI)** |
| --- | --- | --- |
| **Median score imputation** ^a^ | | |
| 6-Minute Walking Distance (m) | p=0.560 | -1.56 (-65.93 to 59.95) |
| Functional Independence Measure (18-126) | p=0.376 | 6.61 (-7.82 to 19.90) |
| **Worst score imputation** ^b^ | | |
| 6-Minute Walking Distance (m) | p=0.526 | -1.39 (-61.29 to 59.37) |
| Functional Independence Measure (18-126) | p=0.270 | 8.09 (-6.28 to 21.92) |

^a^ Median-score imputation (median from both groups) for patients with an unexpected hospital discharge and worst-score imputation for patients that died. One patient in the control group ultimately died before hospital discharge, but since the 6MWD and FIM had already been conducted, the patient’s values were carried forward. Non-normally distributed data, analysed with Mann-Whitney U test. Group difference (experimental group minus control group value) are in means using bias-corrected-and-accelerated bootstrap sampling to estimate 95% Confidence Intervals (95% CI) in case of skewed data.

^b^ Worst-score imputation for patients that died or had an unexpected hospital discharge. One patient in the control group ultimately died before hospital discharge, but since the 6MWD and FIM had already been conducted, the patient’s values were carried forward. Non-normally distributed data, analysed with Mann-Whitney U test. Group difference (experimental group minus control group value) are in means using bias-corrected-and-accelerated bootstrap sampling to estimate 95% Confidence Intervals (95% CI) in case of skewed data.

**Sensitivity analyses for secondary outcomes (n=115):**

There were no significant differences at ICU discharge for imputed FIM (mean difference (95% CI) -0.38 (-6.33 to 5.58); p=0.902) and MRC-sum-score (mean difference (95% CI -2.24 (-7.03 to 2.55); p=0.358).
