# Supplementary material for: Effects of early, combined endurance and resistance training in mechanically ventilated, critically ill patients: A randomised controlled trial
Source: PLoS One. 2018 Nov 14;13(11):e0207428. doi: 10.1371/journal.pone.0207428 (PMC6235392; doi:10.1371/journal.pone.0207428)
Supplement: S3 Table — (PDF) [file pone.0207428.s005.pdf]

S4 Table. Patient characteristics for 'with versus without SF-36' (mental health component) including patients that died.

Variable	with SF-36 (n=61)	without SF-36 (n=54)	p value
Age, years, mean (SD)	68 (IQR 20)	67 (IQR 19)	p = 0.749
Sex, female, n (%)	20 (53%)	18 (47%)	p < 0.999
Weight, kg, mean (SD)	81 ±18	81 ±17	p = 0.828
BMI, kg/m², mean (SD)	26 (8)	27 (IQR 5)	p = 0.953
APACHE II score, mean (SD) [a]	21 (IQR 9)	25 (IQR 10)	p = 0.059
TISS-28 score, median (IQR)	37 (IQR 13)	37 (IQR 11)	p = 0.779
TISS-76 score, median (IQR)	48 (IQR 16)	46 (IQR 18)	p = 0.419
ICU days until study inclusion, mean (SD)	1.7 (IQR 1.7)	1.8 (IQR 1.5)	p = 0.966
SOFA score, median (IQR) [b]	8 (IQR 4)	9 (IQR 5)	p = 0.088
LOS in hospital (days)	24.9 (IQR 29.8)	21.5 (IQR 18.6)	p = 0.062
LOS in ICU (days)	6.2 (IQR 9.6)	6.4 (IQR 9.5)	p = 0.579
Time on mechanical ventilation (days)	6.0 (IQR 9.3)	5.1 (IQR 8.1)	p = 0.477

[a] *at ICU admission*

[b] *at study inclusion*

Data are presented as median (IQR), mean (SD) or n (%)

Abbreviations: *BMI = Body Mass Index, APACHE = Acute Physiology and Chronic Health Evaluation, TISS = Therapeutic Intervention Scoring System, SOFA = Sequential Organ Failure Assessment, CRP = C-reactive protein, LOS = length of stay, ICU = intensive care unit*
