# Supplementary material for: Effects of early, combined endurance and resistance training in mechanically ventilated, critically ill patients: A randomised controlled trial
Source: PLoS One. 2018 Nov 14;13(11):e0207428. doi: 10.1371/journal.pone.0207428 (PMC6235392; doi:10.1371/journal.pone.0207428)
Supplement: S4 Table — (PDF) [file pone.0207428.s006.pdf]

S2 Table. Patient characteristics for 'with versus without resistance training' (RT) regardless of prior group allocation.

**Variable**	**with RT (n=7)**	**without RT (n=108)**	**p value**
Age, years, mean (SD)	71 (IQR 13)	67 (IQR 21)	p = 0.437
Sex, female, n (%)	4 (57%)	34 (32%)	p = 0.217
Weight, kg, mean (SD)	73 ±15	81 ±17	p = 0.187
BMI, kg/m², mean (SD)	27 (10)	26 (IQR 6)	p = 0.888
APACHE II score, mean (SD) [a]	26 (IQR 7)	22 (IQR 10)	p = 0.539
TISS-28 score, median (IQR)	33 (IQR 13)	37 (IQR 12)	p = 0.320
TISS-76 score, median (IQR)	52 (IQR 19)	47 (IQR 16)	p = 0.558
ICU days until study inclusion, mean (SD)	1.8 (IQR 2.1)	1.8 (IQR 1.7)	p = 0.952
SOFA score, median (IQR) [b]	9 (IQR 7)	8 (IQR 5)	p = 0.192
LOS in hospital (days)	49.1 (IQR 66.2)	23.6 (IQR 21.5)	p = 0.171
**LOS in ICU (days)**	**26.3 (IQR 35.0)**	**6.0 (IQR 7.8)**	**p = 0.010**
**Time on mechanical ventilation (days)**	**25.0 (IQR 31.3)**	**5.1 (IQR 7.6)**	**p = 0.011**

[a] *at ICU admission*

[b] *at study inclusion*

Data are presented as median (IQR), mean (SD) or n (%)

Abbreviations: *BMI = Body Mass Index, APACHE = Acute Physiology and Chronic Health Evaluation, TISS = Therapeutic Intervention Scoring System, SOFA = Sequential Organ Failure Assessment, CRP = C-reactive protein, LOS = length of stay, ICU = intensive care unit*
