# Supplementary material for: Effects of early, combined endurance and resistance training in mechanically ventilated, critically ill patients: A randomised controlled trial
Source: PLoS One. 2018 Nov 14;13(11):e0207428. doi: 10.1371/journal.pone.0207428 (PMC6235392; doi:10.1371/journal.pone.0207428)
Supplement: S1 File — (PDF) [file pone.0207428.s007.pdf]

S2 Fig. Physiological parameters while on mechanical ventilation (with indirect calorimetry) for before, during and after physiotherapy.

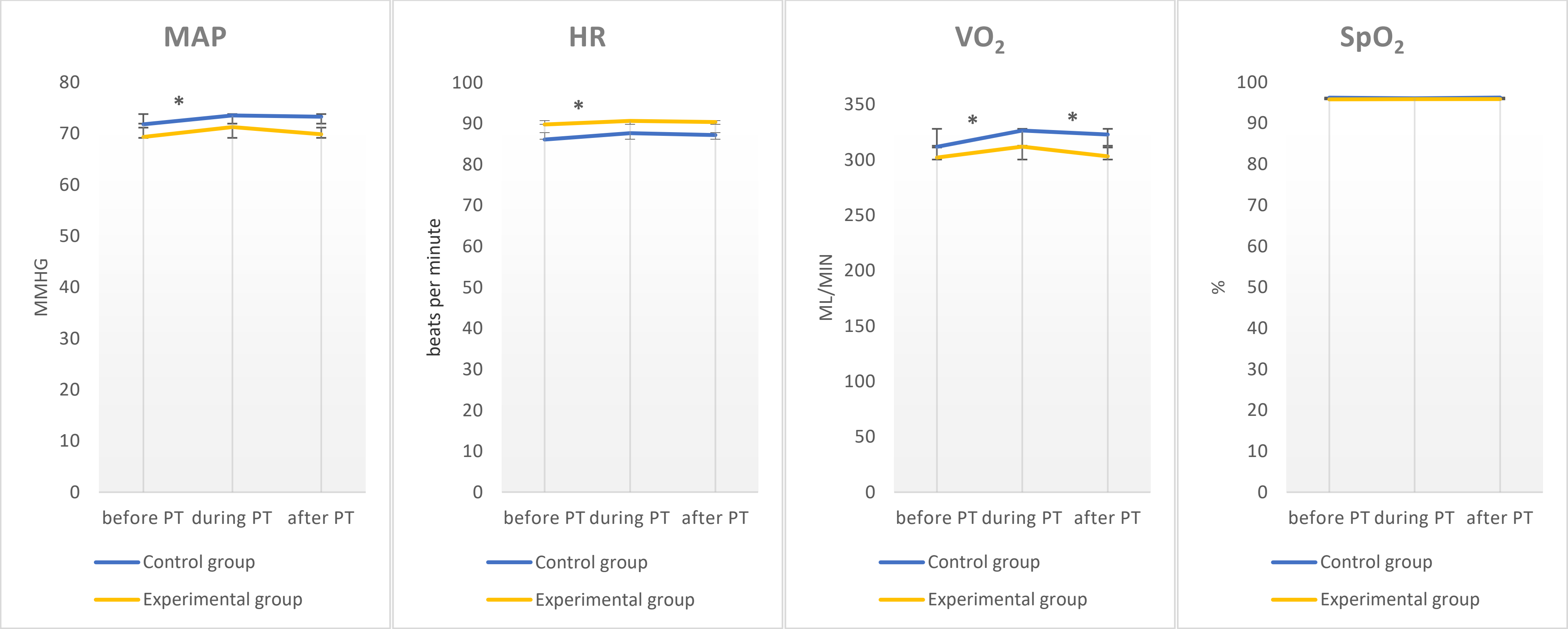

* $p < 0.013$

Only full data sets (from before to after physiotherapy) were included. Total sessions: MAP: experimental n=193, control n=214, HR: experimental n=195, control n=222, VO2: experimental n=166, control n=192, SpO2: experimental n=195, control n=221.

Abbreviations: PT = physiotherapy, MAP = mean arterial pressure, HR = heart rate, VO2 = oxygen consumption, SpO2 = peripheral oxygen saturation

S3 Fig. Physiological parameters for predominantly spontaneously breathing patients for before, during and after physiotherapy.

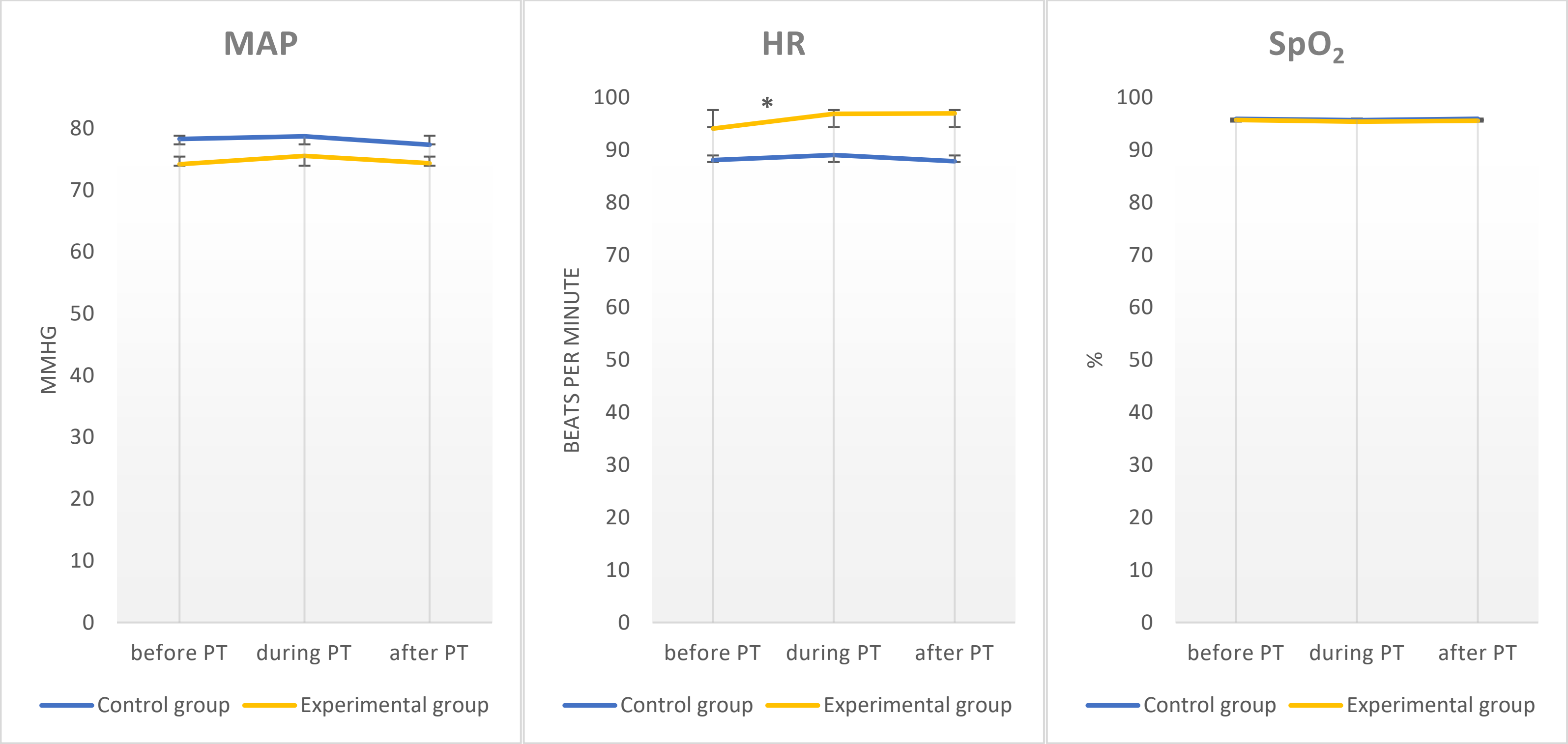

* p = 0.006

Predominantly spontaneously breathing patients included patients with no indirect calorimetry in case of high fiO2, physiotherapy during spontaneous breathing trials or with extubated subjects, second session per day. Only full data sets (from before to after physiotherapy) were included. Total sessions: MAP: experimental n=147, control n=118, HR: experimental n=161, control n=134, SpO2: experimental n=159, control n=134.

Abbreviations: PT = physiotherapy, MAP = mean arterial pressure, HR = heart rate, VO2 = oxygen consumption, SpO2 = peripheral oxygen saturation
