# Supplementary material for: Effects of early, combined endurance and resistance training in mechanically ventilated, critically ill patients: A randomised controlled trial
Source: PLoS One. 2018 Nov 14;13(11):e0207428. doi: 10.1371/journal.pone.0207428 (PMC6235392; doi:10.1371/journal.pone.0207428)
Supplement: S2 File — (PDF) [file pone.0207428.s008.pdf]

## S2 File. Detailed information on performed study interventions: sessions, mobilisations and achieved mobility-milestones

**S4 Fig. Number of patients from each group in increasing quantities of sessions.**

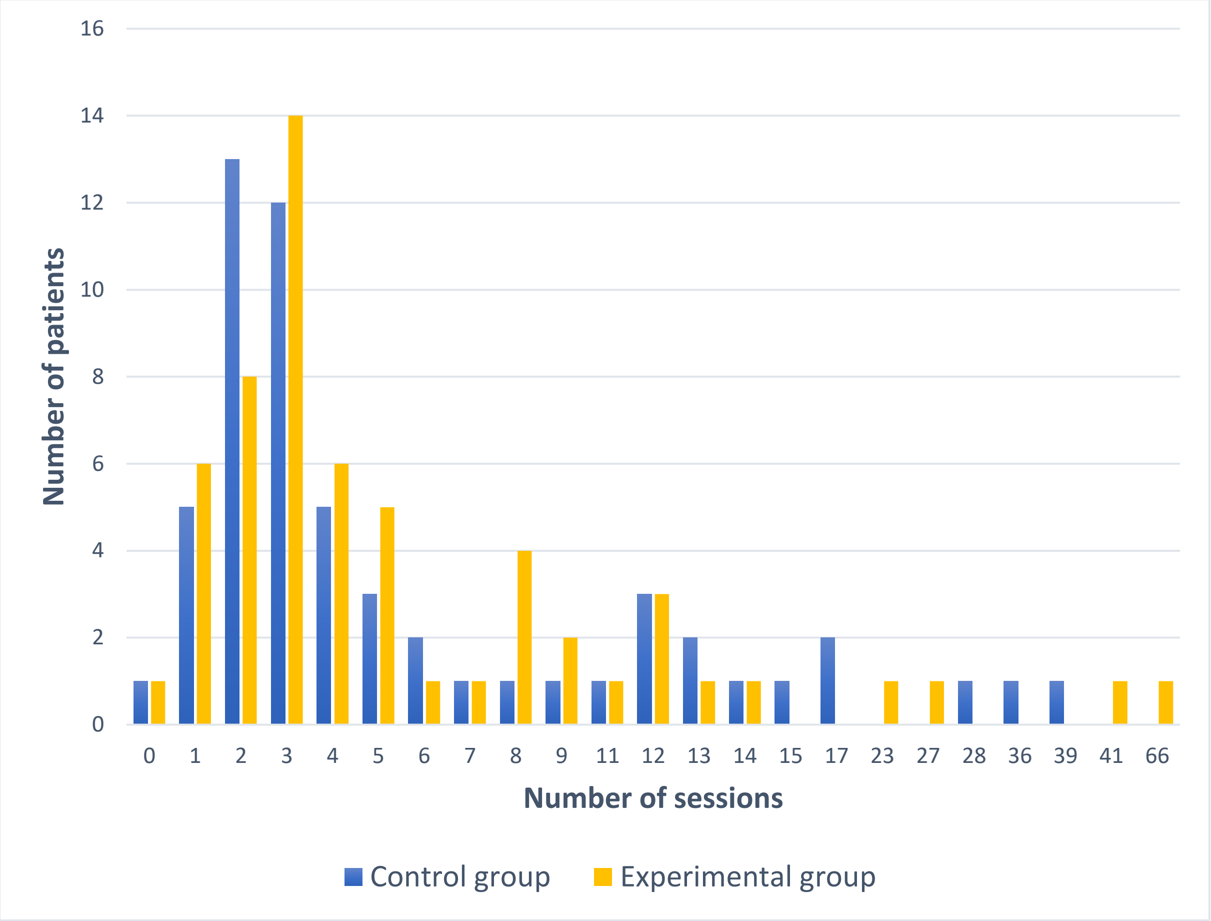

**S5 Fig. Details of physiotherapy-initiated mobilisations for the control and experimental groups.** Includes only physiotherapy-initiated mobilisations on study days.

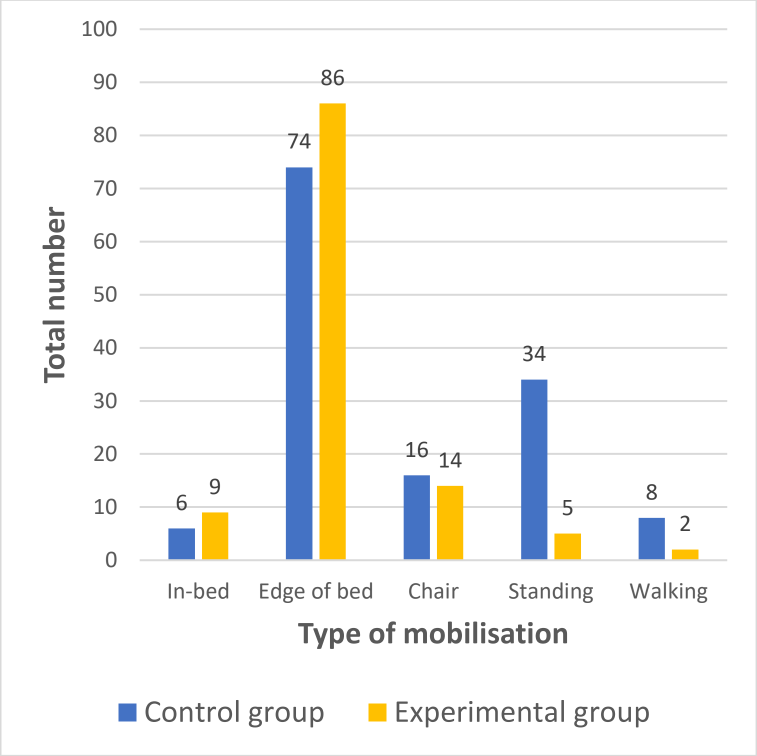


**S6 Fig. Details of all ICU mobilisations for the control and experimental groups.** Includes all mobilisations conducted by various ICU staff as part of ICU standard care during overall ICU stay (including physiotherapy-initiated mobilisations and ICU days before study enrolment).

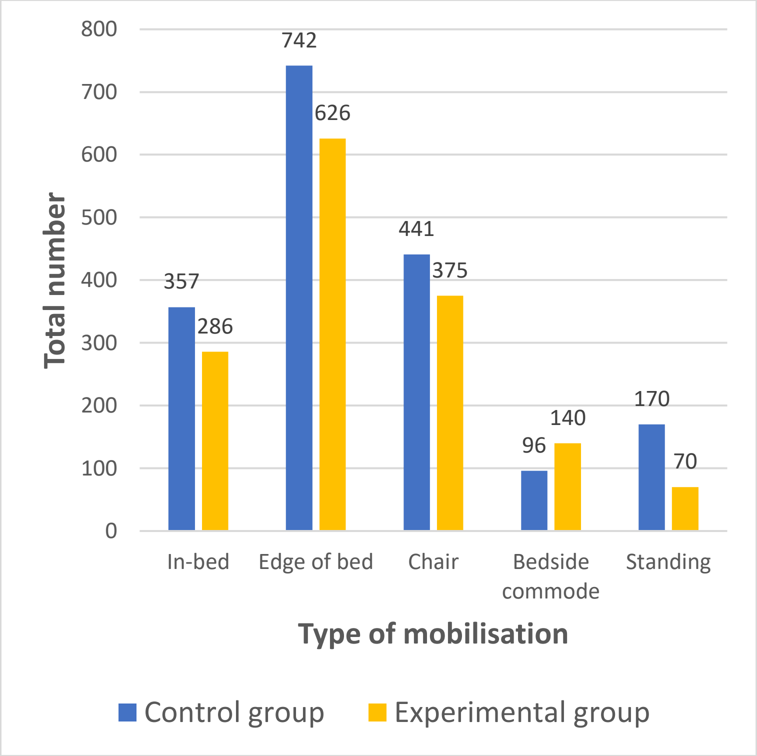

**S7 Fig. Number of achieved mobility-milestones for the control and experimental groups.**

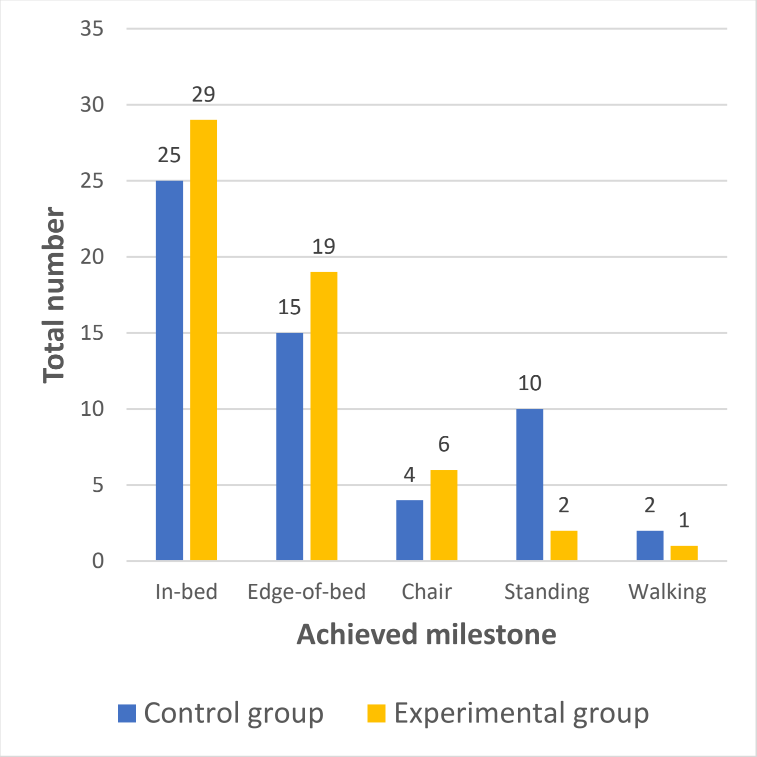


**S5 Table. Time until first mobility-milestone was achieved.**

| | Experimental group n = 58 | | Control group n = 57 | | p value |
|---|---|---|---|---|---|
| | **n** | **value** | **n** | **value** | |
| Time to first edge-of-bed (days) | 24 | 4 (IQR 2-7) | 28 | 5 (IQR 2-7) | p = 0.451 |
| Time to first chair-transfer (days) | 7 | 12 (IQR 5-28) | 10 | 11 (IQR 3-20) | p > 0.999 |
| Time to first standing (days) | 3 | 10 | 12 | 7.5 (IQR 3-14) | - |
| Time to first walking (days) | 1 | 8 | 2 | 23 | - |

Data are presented as median (IQR if n > 10)
