# Supplementary material for: Effects of early, combined endurance and resistance training in mechanically ventilated, critically ill patients: A randomised controlled trial
Source: PLoS One. 2018 Nov 14;13(11):e0207428. doi: 10.1371/journal.pone.0207428 (PMC6235392; doi:10.1371/journal.pone.0207428)
Supplement: S3 File — This protocol has been approved by the local ethics committee prior to first patient recruitment. (PDF) [file pone.0207428.s009.pdf]

Effects of early, combined endurance and resistance training in mechanically ventilated, critically ill patients: a randomised controlled trial

Sabrina Eggmann[1*], Martin L Verra[1], Gere Luder[1], Jukka Takala[2], Stephan M Jakob [2]

[1]Department of Physiotherapy, Inselspital, Bern University Hospital, Bern, Switzerland

[2]Department of Intensive Care Medicine, Inselspital, Bern University Hospital, University of Bern, Switzerland

S3 File. Original study protocol and English translation.

Page 2-16: Original study protocol
Page 17-31: English translation

Studienprotokoll

Auswirkungen eines frühen Ausdauer- und Krafttrainings beim beatmeten kritisch Kranken auf der Intensivstation - eine randomisierte kontrollierte Studie.

1. Titel des Studienprotokolls

1.1 Titel: Auswirkungen eines frühen Ausdauer- und Krafttrainings beim beatmeten kritisch Kranken auf der Intensivstation - eine randomisierte kontrollierte Studie.

1.1.1 Version 3 vom 17. September 2012 (KEK-BE: 122/12, DLF Studie: 2221, UTN (University Trial Number): U1111-1134-4362, Registrierungnr.: DRKS00004347)

1.1.2 Unterschrift Prüfer Prof. Dr. med. Stephan Jakob: Bern, den 17.9.12

____

3. Zielsetzungen und Zweck

3.1 Hintergrund, Begründung und Ziel der Studie

Auf der Intensivstation mit dem Hauptfokus des Wiederbelebens und Überlebens liegt herkömmlicherweise wenig frühe Aufmerksamkeit auf den neuromuskulären Funktionen. Die kritische Erkrankung, häufig Sepsis und Multiorganversagen, steht im Vordergrund. In den 80er Jahren beschrieben Bolton et al. bei Patienten mit Zustand nach Sepsis eine unerklärliche verlängerte Entwöhnung der Beatmung, verminderte Muskeleigenreflexe, sowie eine generalisierte Muskelschwäche. Sie nannten dieses Phänomen Critical Illness Polyneuropathie. [Bolton, 1986]

Heute gibt es klare Definitionen und diagnostische Kriterien für dieses Phänomen. Die „erworbene Skelettmuskelschwäche bei kritisch Kranken" („ICU-acquired weakness", ICUAW) beschreibt dabei eine rein klinisch diagnostizierte, neu aufgetretenem diffuse Muskelschwäche bei Intensivpatienten ohne bestimmbare Ursache bis auf die kritische Erkrankung selbst und einem Medical Research Council Summenscore von < 48. [Stevens, 2009]

Die erworbene Skelettmuskelschwäche beim kritisch Kranken auf der Intensivstation ist mit einem schlechten Outcome assoziiert. Einerseits wird durch die ICUAW die Beatmungszeit bis zu 2,5mal länger. [De Jonghe, 2004] Weiter leiden mehr als 30% der Überlebenden ein Jahr nach ihrem Intensivstationsaufenthalt unter einem schlechten funktionellen Status. Die Patienten sind dabei vor allem in ihrem sozialem Verhalten und ihrer Mobilität eingeschränkt. Die Lebensqualität ist stark beeinträchtigt. [Van der Schaaf, 2009]

Allein Bettruhe und Immobilisation können bereits schwere Komplikationen verursachen, zum Beispiel Skelettmuskelatrophie und Schwäche, Gelenkkontrakturen, Atelektasen, Dekubitus, Thrombosen, systemische Entzündung, etc. Gegenmassnahmen sind unter anderem Widerstandsübungen, passive Dehnübungen und Mobilisation. [Brower, 2009]

Physiotherapeuten mit ihrem spezifischen Fachwissen über neurologische und muskuloskelettale Funktionen könnten im Umfeld der Intensivstation eine wesentliche Rolle spielen. Durch ihr Fachwissen erreichen sie oft höhere Mobilitätsgrade als andere Gesundheitsberufe. [Garzon-Serrano, 2012]. Ausserdem zeigen neue Studien, dass frühe Mobilisation und Physiotherapie auf der Intensivstation nicht nur sicher und machbar, sondern auch mit einem besseren Spitaloutcome assoziiert ist. [Morris, 2008, Burtin,2009, Schweickert, 2009]. Durch eine frühe Mobilisation verkürzte sich der Aufenthalt auf der

Intensivstation und im Spital signifikant. [Morris, 2008]. Ein frühes Fahrradtraining verbesserte die Leistungsfähigkeit (gemessen mit dem 6 min Gehtest) und die Lebensqualität (gemessen mit dem Fragebogen SF-36). [Burtin, 2009] Weiter erreichten Patienten mit Physiotherapie einen höheren Level bei Alltagsaktivitäten und verbrachten weniger Tage mit Delirium. [Schweickert, 2009]

Trotzallem besteht gemäss der "European Respiratory Society and European Society of Intensive Care Medicine Task Force" lediglich eine Evidenz vom Level C-D für Physiotherapie auf der Intensivstation. [Gosselink, 2008]. Ebenfalls fehlt eine generelle Einigung oder eine Evidenz-basierte Leitlinie zu Intensität, Frequenz, optimaler Beginn und Dauer der Therapie. [NICE guidelines, 2009] Sehr unklar ist die Evidenz zur Behandlung der Gelenkbeweglichkeit bei sedierten Patienten. [Stockley, 2012] Ferner wurden die meisten obengenannte Studien auf einer respiratorischen oder medizinischen Intensivstation durchgeführt. [Morris, 2008, Schweickert, 2009, Pohlman, 2010] Dementsprechend fehlen Daten mit chirurgischen oder neurologischen Patienten auf der Intensivstation. Weitere randomisierte kontrollierte Studien sind also nötig, um mögliche kurz- und langfristige Nutzen zur Muskelkraft, physische Funktionen und Lebensqualität der Patienten zu evaluieren und die Nützlichkeit und Wirksamkeit von Interventionen z.B. Mobilisationshilfen oder Fahrradergometer festzustellen. [Fan, 2009]

Dementsprechend ist das Ziel dieser Studie auf einer interdisziplinären Intensivstation ein frühes Mobilisationskonzept mit einem Ausdauer- und Krafttraining zu kombinieren.

Dabei soll in erster Linie die Durchführbarkeit und Sicherheit mit einem gemischten Patientengut festgestellt werden und als primäres Outcome die Leistungsfähigkeit und Selbständigkeit im Alltag gemessen werden. Als sekundäres Outcome werden die Kraft, Beweglichkeit, und die Lebensqualität gemessen. Variablen zur Sicherheit der Behandlung sind der Sauerstoffverbrauch, die Oxygenation und die Hämodynamik während der Behandlung.

Durch die geplante Studie erwarten wir neue Kenntnisse zur Sicherheit und Durchführung von früher Physiotherapie auf der Intensivstation und einen Wissensgewinn zu deren kurzzeitigen Nutzen, nebst der Leistungsfähigkeit vor allem auch die Auswirkung auf die Beweglichkeit und die Kraft, welche wichtige Voraussetzungen sind, um Alltagsaktivitäten durchzuführen. Zudem kann uns der Sauerstoffverbrauch in Ruhe und Anstrengung beim beatmeten Patienten wichtige Daten zu Sicherheit der Therapiedauer und Intensität liefern.

Das Studienthema ist klinisch relevant. Falls die physiotherapeutischen Massnahmen der Interventionsgruppe die der Kontrollgruppe („usual care") überlegen sein würden, wird die Implementierung der neuen Behandlungstechniken im klinischen Alltag relativ einfach sein. Damit ist das Projekt generalisierbar und extern valide. Es wird durch Prof. Dr. med. Jukka Takala, Direktor und Chefarzt der Universitätsklinik für Intensivstation und von Martin L. Verra, MPtSc., Direktor des Instituts für Physiotherapie unterstützt und getragen.

3.2 Fragestellungen, Studienpopulation

3.2.1 Hauptsächliche Fragestellung:

- Erreichen die Patienten mit einem angepassten Ausdauer- und Krafttraining auf einer interdisziplinären Intensivstation eine höhere Leistungsfähigkeit und Selbständigkeit im Alltag bei Spitalaustritt?

3.2.2 Weitere Fragestellungen:

- Ist eine frühe Mobilisation mit angepassten Ausdauer- und Krafttraining auf einer interdisziplinären Intensivstation sicher und durchführbar?
- Kann eine frühe Mobilisation und Training die Kraft und Beweglichkeit bereits bei Intensivstationsaustritt verbessern?
- Besteht ein direkter Zusammenhang zwischen den Komponenten Beweglichkeit und Kraft mit der Selbständigkeit im Alltag?
- Besteht ein Zusammenhang zwischen den Outcomedaten und den Komplikationen von Bettruhe (Schwäche/ ICUAW, Kontraktur, Atelektase und Dekubitus, Pneumonie, Thrombose und Delir)?
- Haben Patienten der Behandlungsgruppe 6 Monate nach Spitalaustritt eine höhere Lebensqualität?

3.2.3 Studienpopulation:

Untersucht werden stationäre Patienten der Universitätsklinik für Intensivmedizin, welche eine erwartete Beatmungszeit von mindestens 72h auf der Intensivstation haben.
Da der Muskelabbau bereits in den ersten Tagen der Bettruhe beginnt soll die physiotherapeutische Intervention so früh als möglich beginnen. Daher wird angestrebt die, für die Komplikationen der Bettruhe gefährdeten, Patienten möglichst früh zu entdecken und zu behandeln. Entsprechend wurde der Einschluss bei einer erwarteten Beatmungszeit von mindestens 72h gewählt.

3.3 Hypothesen

- Eine frühe Mobilisation kombiniert mit einem angepassten Ausdauer- und Krafttraining auf einer interdisziplinären Intensivstation ist sicher und machbar. Dabei verbessert sich die Leistungsfähigkeit dieser Patienten, was im Alltag zu mehr Selbständigkeit führt.
- Eine frühe Mobilisation kombiniert mit einem angepassten Ausdauer- und Krafttraining auf einer interdisziplinären Intensivstation kann die Kraft und Beweglichkeit verbessern. Die Lebensqualität nach 6 Monaten verbessert sich durch das frühbeginnende Training.

4. Studiendesign

4.1 Hauptzielparameter, und sekundäre Zielparameter

- Hauptzielparameter sind die Leistungsfähigkeit und die Selbständigkeit im Alltag bei Spitalaustritt.
- sekundäre Zielparameter sind die Kraft, die Beweglichkeit und die Selbständigkeit im Alltag bei Intensivstationsaustritt, sowie die Lebensqualität 6 Monate nach Spitalaustritt.
- weitere Zielparameter: Anzahl Tage auf der Intensivstation und im Spital

4.2 Studiendesign

- Randomized Controlled Trial, prospektiv mit Follow-up nach 6 Monaten
- Hauptstudie (Phase 2 Studie, d.h. Pilot RCT mit Fokus auf Durchführbarkeit und preliminary effectiveness – Zwischenresultate werden für die MSc These von Sabrina Eggmann an der Donau-Universität Krems, Lehrgang: Master of Science in Neurorehabilitation, veröffentlicht)
- Interventionsstudie mit Verblindung des Statistikers und Assessors
- Monozentrisch
- National

4.2.1 tabellarische Übersicht der Untersuchungen mit Zeitraster (Flowchart)

Untersuchung	Zeitpunkt
**Verlaufskontrollen**	Täglich während der Intervention
**Sicherheitskontrollen**	Täglich während der Intervention
**Kraftmessung**	Tag 7, 21, 35,… Austritt Intensivstation
**Functional Independece Measure**	Tag 7, 21, 35,… Austritt Intensivstation / Spital
**Messung der Beweglichkeit**	Tag 7 und Austritt Intensivstation
**6-Minuten-Gehtest**	Spitalaustritt
**Timed-Up-An-Go**	Spitalaustritt
**Fragebogen SF-36**	6 Monate nach Spitalaustritt

4.2.2 Studienablauf

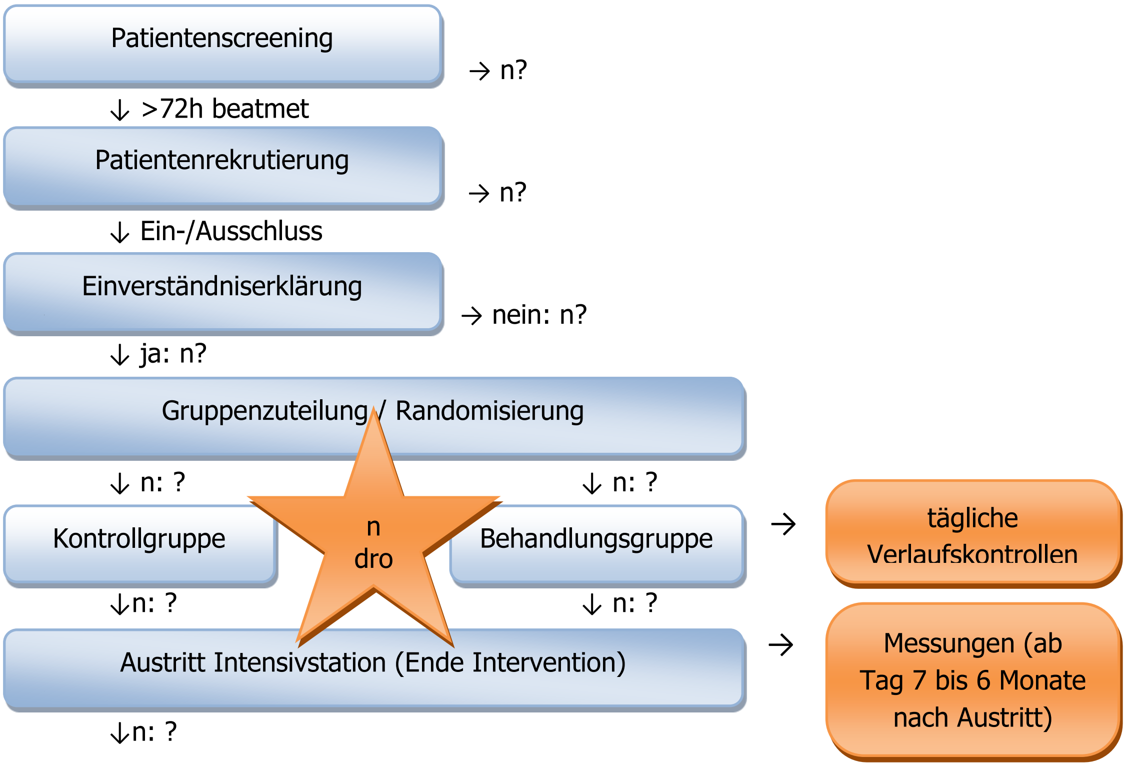

4.2.3 Interventionen

4.2.3.1 Kontrollgruppe

- Beginn der Physiotherapie sobald Verordnung des behandelnden Arztes, tägliche Abklärung durch Physiotherapeut (PT)
- Atem- oder Schlucktherapie nach Bedarf
- Physiotherapie 5-7x wöchentlich, Entscheid durch behandelnde PT, gemäss bestehendem Protokoll
- Physiotherapie 1x täglich (bei zusätzlicher Schlucktherapie, 2x täglich)
- sedierte Patienten: passive Bewegungstherapie mit 3-10 Wiederholungen (Whg), jedes Gelenkes, in alle möglichen Richtungen der jeweiligen Patientenposition, Lagerung gemäss bestehendem Dekubitusprotokoll

- wache Patienten: passive, aktiv-assistiert oder aktive Bewegungstherapie, je nach Möglichkeit des Patienten (Entscheid durch behandelnder PT) und Lagerung
- Mobilisation beginnend an Bettrand, nach Verordnung des behandelnden Arztes

4.2.3.2 Interventionsgruppe

Allgemeines:
- Beginn der Physiotherapie sobald Verordnung des behandelnden Arztes, tägliche Abklärung durch PT
- Atem- oder Schlucktherapie nach Bedarf
- Physiotherapie 5-7x wöchentlich, Entscheid durch behandelnde PT, gemäss bestehendem Protokoll
- Physiotherapie 1x täglich (Level I-II), 1-2x täglich (Level III), 2x täglich (Level IV-VI), max. 2x täglich (inklusive Schlucktherapie)
- Mobilisation beginnend im Bett, nach Verordnung des behandelnden Arztes
- falls möglich Reduktion der Sedativa für Therapie (nach Verordnung des behandelnden Arztes: Level II)

Therapieübersicht:

Level I	Level II	Level III	Level IV	Level V	Level VI
Sediert	Sedations-stopps	Mobi freigegeben	10min BR	50% Hilfe Transfer, M3 4ceps	50% Hilfe Stand
p ROM	p / a.ass / a ROM	p / a.ass / a ROM	p / a.ass / a ROM	a.ass / a ROM	a.ass / a ROM
Lagerung	Aufrecht Bett	Bettrand	Lehnstuhl	Stand	Gehen
MOTOmed Letto 2 (Bettfahrradergometer) Training					

p = passive, a.ass = aktiv - assistierte, a = aktive, ROM = Bewegungstherapie (Range of Motion)

Bewegungstherapie und Krafttraining:
- pROM: mit 3-10 Whg, jedes Gelenkes in alle möglichen Richtungen der jeweiligen Patientenposition, falls möglich mit Einbezug von Alltagsaktivitäten (ADL).
- a.ass. ROM: mit so viel Unterstützung wie nötig zur Erreichung des vollen Bewegungsausmasses, 3-10 Whg, jedes Gelenkes in alle möglichen Richtungen der jeweiligen Lage, falls möglich ADL Einbezug.
- spezifische Beweglichkeitsübungen für Hände und Füsse als Heimprogramm → ein entsprechendes Blatt hängt bei Patientenbett.
- a ROM / Krafttraining: sobald a.ass. ROM mit < 25% Hilfe durchführbar ist werden die Übungen mit Gewicht (Start bei 450g, 1kg, 2kg, etc.) oder therapeutischem Widerstand ausgeführt. Die therapeutische Führungshilfe dient vor allem zur Erreichung des vollen Bewegungsausmasses und der qualitativ korrekten Durchführung.
- Muskelgruppen für das Krafttraining: Hüftgelenksabduktoren, Kniegelenksextensoren, Fussdorsalextensoren, Schultergelenkflexoren und -Aussenrotatoren, Ellenbogenflexoren (zusätzliches individuelles Training nach Entscheid PT, z.B. Rumpfmuskulatur, Hüftgelenksextensoren)

- Trainiert wird auf einer mittleren Belastungsintensität (50-70%) mit 8-12 Whg und 2-5 Serien, zwischen den Serien erfolgen mindestens 2 Minuten Pause
- Jeweils montags, mittwochs und freitags: Krafttraining für obere Extremität und entsprechend dienstags und donnerstags: untere Extremität
- Sobald ein Patient die Kraftübungen korrekt durchführt, darf er diese mit den Angehörigen oder der Pflege tagsüber üben. Das Übungsprogramm wird in diesem Fall in der Nähe des Patientenbettes aufgehängt.

Ausdauertraining mit MOTOmed Letto2, Bettfahrradergometer:
- Intensität: 1x täglich, 5x wöchentlich
- Ausgangsstellung des Patienten: Rückenlage mit 30° erhöhtem Kopfteil
- sedierte Patienten: passiv, bis zu 20 min und mit maximal 20 U/min
- gemischt aktiv - passiv tretende Patienten: bis zu 20 min, 20 U/min, 0 Grad Widerstand
- 20 min aktiv tretende Patienten (Grad 0): Erhöhung des Widerstandes
- 20 min aktiv tretende Patienten (Grad 2): Steigerung auf 30 min, anschliessend Widerstanderhöhung um 1 Grad jeden 2. Tag, bis Grad 6
- 30 min aktiv tretende Patienten (Grad 6): Steigerung der Zeit bis max. 60min
- individuelle Anpassungen / Steigerungen möglich (Entscheid PT)

Mobilisation mit ADL Einbezug:
- Level I: sedierte Patienten: Lagerung gemäss Dekubitusprotokoll
- Level II: Sedationsstopps: Aufrechte U-Lagerung im Bett
- Level III: Bettrand, Lagerung individuell (z.B. mit Rückenlehnen, Armstützen, etc.)
- Level IV: Lehnstuhl, Transfer individuell (z.B. tief mit Leintuch, tief ohne Leintuch, hoch via Stand → Entscheid PT), Voraussetzung: mind. 10min Bettrand möglich
- Level V: Stand (mit Hilfsmittel oder Hilfsperson → PT / Pflegefachperson), Voraussetzungen: Kraft von M3 im M. Quadriceps, < 50% Unterstützung beim Transfer
- Level VI: Gehen (mit Hilfsmittel oder Hilfsperson → PT / Pflegefachperson), Voraussetzungen: <50% Unterstützung im Stand, Schritte machen an Ort möglich
- ADL wird individuell angepasst und einbezogen, Entscheid durch behandelnde PT (z.B. drehen im Bett, bridging, Gleichgewicht im Sitz / Stand, sit-to-stand, Transfer, etc.)

4.2.4 Studiendauer pro Patient
Die Intervention findet während des Intensivstationsaufenthaltes statt, dementsprechend ist die Studiendauer für jeden Patienten individuell. Die letzte Messung findet 6 Monate nach Spitalaustritt in Form eines Fragebogens statt.

4.2.5 Abbruchkriterien
Grosse Schwankungen in Puls, Blutdruck oder Sauerstoffsättigung während der Behandlung, oder eine starke Zunahme des Sauerstoffverbrauchs können zu einem Studienabbruch führen. Die Entscheidung zum Abbruch liegt beim Studienarzt.
Eine Therapiesitzung kann durch den Therapeuten abgebrochen werden (siehe definierte Abbruchkriterien 7.1.1). In diesem Fall wird die Therapie erst am nächsten Tag wieder aufgenommen.

4.2.6 Bedingungen für Entblindung

Der Patient und das Behandlungsteam sind nicht verblindet.

4.3 Massnahmen zur Bias-Minimierung

- Randomisierung
- Verblindung der Statistiker und Assessoren
- die medizinische Behandlung bleibt bei beiden Gruppen gleich

5. Auswahl von Versuchspersonen

5.1 Rekrutierung

Rekrutierung der Studienteilnehmer: Screening durch Forschungsteam der Intensivstation mittels Patient Data Management System (PDMS)

Bereitschaft zur Teilnahme: Deferred Consent (innerhalb 72h laut Empfehlung der AGEK), vorliegende mutmassliche Willensäusserung durch Familienangehörige oder eine unterschriebene Einwilligungserklärung des Patienten falls möglich, eingeholt durch den behandelnden Arzt.

5.2 Einschlusskriterien

- ≥ 18 Jahre
- erwartete Beatmung von > 72h auf interdisziplinären Intensivstation
- qualitativ selbständig vor Erkrankung

5.3 Ausschlusskriterien

- Kontraindikation für Bettfahrradergometer MOTOmed (instabile Frakturen/ offene Wunden in UE/ Becken/ Bauch)
- Gewicht > 150kg
- vorbestehende neuromuskuläre / neurologische Erkrankung, die zu bleibenden Einschränkungen der Kraft, Beweglichkeit, Leistungsfähigkeit oder Selbständigkeit im Alltag führten)
- vorheriger Spitalaufenthalt von mehr als 10 Tagen
- Therapielimitierung wegen erwartetem schlechten Outcome
- Einschluss in andere Interventionsstudie
- Eintrittsdiagnosen, welche einen Gehtest nach Akutstadium verunmöglichen: z.B. Patienten mit ausgeprägtem Cerebro Vaskulärem Insult, schwerem Schädelhirntrauma, Rückenmarksverletzung mit Tetra- oder Paraplegie, etc.

6. Bewertung der Wirksamkeit

6.1 Wirksamkeitsparameter: Messmethoden und Zeitpunkte

6.1.1 Hauptzielparameter

- Leistungsfähigkeit: 6-Minuten-Gehtest (6MWT) und Timed-Up-And-Go (TUG) bei Spitalaustritt, gemessen durch Physiotherapeuten (PT)
- Selbständigkeit im Alltag: Functional Independence Measure (FIM) bei Austritt Intensivstation und Spitalaustritt, ausgefüllt durch das Behandlungsteam (Pflege und PT)
- Meilensteinerreichung während des Intensivstationsaufenthaltes in Anzahl Tagen für Mobilisationniveau (Bettrand / Lehnstuhl / Stand / Gehen)

6.1.2 sekundäre Zielparameter

- Kraft, Testungen ab Tag 7 (Verlaufskontrollen alle 14d) und bei Austritt Intensivstation, durch Physiotherapeuten, Voraussetzung: Patient befolgt 3 von 5 Befehlen:
  - Medical Research Council sum score (MRC) für 6 Muskelgruppen
  - Handkraft mittels JAMAR Dynamometer
  - Quantitativer Muskeltest für Kniegelenksextensoren mittels Kraftmessgerät (Hand Held Dynamometer)
- Beweglichkeit (Range of motion - ROM) mit Goniometer nach Neutral-Null-Methode, Testungen am Tag 7 und bei Austritt Intensivstation durch Physiotherapeuten
- Lebensqualität: Short Form 36 Gesundheitsfragebogen (SF-36) 6 Monate nach Spitalaustritt
- Anzahl Tage auf der Intensivstation und im Spital

7. Bewertung der Sicherheit

7.1 Sicherheitsparameter: Messmethoden und Zeitpunkte

- kontinuierliches 24h Monitoring der Sauerstoffsättigung, Puls und Blutdruck gemäss Intensivstationsstandard, Datenerfassung im Patient Management System (PDMS)
- Sauerstoffverbrauch (VO2): indirekte Kalorimetrie ½h vor, während und 15 Minuten nach der Behandlung von beatmeten Patienten, durchgeführt von der behandelnder Intensivpflegefachperson, Datenerfassung mit PDMS
- tägliche Erfassung von folgenden Komplikationen: ICUAW, Atelektasen, Pneumonie, Dekubitus, Kontraktur, Thrombose, Delir und Critical Illness Polyneuropathie oder Myopathie
- Erfassung der Anzahl erfolgter Behandlungen

7.1.1 definierte Kriterien zur Unterbrechung oder Abbruch der Therapie:

- subjektive starke Müdigkeit / Anstrengung des Patienten
- ärztlich verordnete Limiten unterschritten / Limiten überschritten (insbesondere Hämodynamik, Oxygenation und Sauerstoffverbrauch)
- Ventilator Asynchronie
- keine Patientenkooperation
- starke Schmerzen
- schädliches Ereignis

7.1.2 Verlaufskontrollen:

- Befunde vor, während und nach Behandlung (falls erhebbar):
  - Richmond Agitation Sedation Scale (RASS)
  - Schmerzen auf Visual Analog Scale (VAS)
  - Belastungsempfinden – BORG Skala
- spezielle Befunde während Behandlung zur Verlaufsdokumentation:
  - Bewegungsausmass
  - Krafttrainingsparameter
  - Ausdauertrainingsparameter
  - Mobilisations- und ADL- Parameter

7.2 Sicherstellung der Nachbeobachtung von Versuchspersonen nach unerwünschten Ereignissen

kontinuierliches 24h Monitoring und Safety Analyse durch PDMS (Patient Data Management System)

Auflistung aller schädlichen Ereignisse während und bis 15 Minuten nach der Behandlung, die neu auftreten und nicht auf Intervention reagieren oder trotz Therapieunterbruch anhalten:

- neue hämodynamisch relevante Rhythmusstörung
- neue Oxygenationsstörung (SpO2 < 85%)
- neue instabile Hämodynamik

weitere schädliche Ereignisse:

- akzidentelle Entfernung eines Tubus, Katheters oder ähnlichem
- Sturz
- sonstige Verletzung

serious adverse event: klinische Beurteilung des Prüfarztes, ob studienrelevant

8. Statistik

8.1 Definition des primären Endpunktes und der sekundären Endpunkte

Der minimale klinisch wichtige Unterschied beim 6-Minuten-Gehtest für Patienten mit chronischer Lungenerkrankung wurde bei 50m validiert. [Redelmeier, 1997, ATS, 2002].

Zum Gruppenvergleich werden folgende Basisdaten der Patienten erhoben:

- Alter, Geschlecht, Gewicht, Body Mass Index (BMI)
- APACHE II (Acute Physiology and Chronic Health Evaluation II)
- SOFA score (Sequential Organ Failure Assessment score)
- TISS (Therapeutic Intervention Scoring System) erste Erfassung nach Eintritt und letzte vor Austritt von Intensivstation
- Medikamente, jeweils Anzahl Tage und tägliche Dosis:
    - Relaxantien
    - Vasopressoren
    - Sedativa
    - Opiate
    - Steroide
    - Insulin
- Laborwerte : CRP (C-Reaktives Protein), Kreatinin, Bilirubin, Thrombozyten (Tc), Leukozyten (Lc), Hämoglobin (Hb) → vorhandene Werte bei Eintritt Intensivstation, nach Studieneinschluss und bei Austritt Intensivstation
- Eintrittsdiagnose und diagnostische Gruppe
- Vorhandensein einer Tracheotomie oder eines Beatmungstubus

8.2 Geplante Anzahl Versuchspersonen mit nachvollziehbarer Begründung (ev. Poweranalyse)

Um eine Differenz von 50m im 6-Minuten-Gehtest mit einer statistischen Power von 80% und einem α von 0.05 zu demonstrieren ist eine Stichprobengrösse von 36 Patienten pro Gruppe nötig. Bei einem erwarteten „drop-out" von 20% und einer erwarteten hohen Korrelation der beiden primären Endpunkte (6-Minuten-Gehtest und FIM) sollen 115 Patienten in die Studie eingeschlossen werden. Im Jahr hat das Inselspital Bern im Schnitt 90 Patienten welche länger als 3 Tage beatmet werden und die Einschlusskriterien erfüllen. Aufgrund vorherigen Studien [Winkelman, 2012] wird einen Einschluss von ca. 74% erwartet (keine invasiven Methoden, Therapie kommt in der Regel gut an.) Dementsprechend ist eine Studiendauer von 19 Monaten vorgesehen: 17. September 2012 – 31. Mai 2014.

8.3 Beschreibung der vorgesehenen statistischen Methoden und der geplanten Zwischenauswertungen

Auswertungen mit dem SPSS Programm:

- Deskriptive Statistik bezüglich des Inhalts
- Vergleich innerhalb und zwischen den Gruppen
- Vergleich der demographischen Daten
- signifikante Unterschiede

Für Gruppenvergleich: students-t-test (Normalverteilung) oder Mann-Withney-U Test (keine Normalverteilung)
Für Proportionenvergleich zwischen den beiden Gruppen: Fishers Exact Test.
Für Repetitive Daten: ANOVA Analyse

Zwischenauswertung der Daten für Masterthese von Sabrina Eggmann im September 2013.

8.4 Geplantes Signifikanzniveau

statistische Power von 80% und ein α von 0.05

8.5 Umgang mit fehlenden Daten sowie mit Daten bei vorzeitigem Studienabbruch von Teilnehmern

- Analyse mittels intention-to-treat und per-Protokoll
- bei vorzeitigem Studienabbruch wird der Patient angefragt, ob die Daten weiterverwendet werden dürfen. Ansonsten werden sie vernichtet.

8.6 Definition der Auswertungsgruppen (Intention To Treat, Per Protocol, etc.)

Analyse mittels intention-to-treat und per-Protokoll

9. Studienspezifische Vorsichtsmassnahmen und Pflichten

Die Patienten haben keine Pflichten und müssen keine Vorsichtsmassnahmen treffen.

10. Pflichten des Prüfers

10.1 Die Studie wird gemäss Protokoll, GCP, und den geltenden gesetzlichen Bestimmungen durchgeführt.

10.2 Von schwerwiegenden unerwünschten Ereignissen, (Protokoll-) Änderungen wird berichterstattet. Zwischen- und Abschlussberichte gehen an Ethikkommission.

10.3 Stellungnahme zur Deckung von Schäden

Durch Versicherung des Inselspitals, Universitätsspital Bern gewährleistet

11. Ethische Überlegungen

11.1 Bewertung des Risiko-Nutzen Verhältnisses

Physiotherapie auf der Intensivstation ist sicher. Die Inzidenz von schädlichen physiologischen Ereignissen ist mit 0.2% sehr tief. [Zeppos, 2007] Schädliche Ereignisse sind unüblich, bei 498 Physio- und Ergotherapiesitzungen gab es bei Schweickert et al. ein versehentlich entfernter Radialartherienkatheter und eine Desaturation des Sauerstoffes auf 80%. [Schweickert, 2009] Bei Morris et al. gab es keine schädlichen Ereignisse. [Morris,

2008] Der Nutzen für die Patienten war bei beiden Studien hoch. So waren die Patienten weniger lange im Bett, kürzer im Spital und auf der Intensivstation bei Morris et al. und verliessen das Spital mit einer höheren Selbständigkeit im Alltag bei Schweickert et al. Ein frühes Beginnen von Physiotherapie bereits in den ersten Phasen einer kritischen Erkrankung verbessert funktionale und psychologische Endpunkte bei beatmeten Patienten [Schweickert, 2009]. Dies ist ein wesentlicher Punkt für die Rückkehr ins Leben und bleibt bei mehr als 30% der Patienten nach einer kritischen Erkrankung beeinträchtigt. [van der Schaaf, 2009] Die Belastung bei Überlebenden einer kritischen Erkrankung ist generell sehr hoch. Dementsprechend sind effektive Therapien dringend gefragt und sollten früh ansetzen. [Hofhuis, 2008]

Am Inselspital Bern ist die Physiotherapie seit langem ein fester Bestandteil der Intensivstation. Die behandelnden Therapeuten sind erfahren und es besteht eine gute interdisziplinäre Zusammenarbeit. Die frühe Mobilisation ist gut etabliert und es gibt selten Zwischenfälle. Die Studie wird von Prof. Dr. med. Jukka Takala, Direktor und Chefarzt der Universitätsklinik für Intensivstation und von Martin L. Verra, MPtSc., Direktor des Instituts für Physiotherapie unterstützt und getragen.

11.2 Beschreibung, warum besonders schützenswerte Versuchspersonen eingeschlossen werden

Da die zuvor erwähnten Studien uns Hinweise geben, dass Therapie früh ansetzen soll und es sicher ist sie schon auf der Intensivstation zu beginnen, wird diese besonders schutzbedürftige Patientengruppe gewählt.

Um dabei möglichst den Willen des Patienten einzubeziehen werden seine Angehörigen innerhalb von 72h nach seinem mutmasslichen Willen befragt. Zusätzlich wird ein von der Studie unabhängiger Arzt befragt, damit mit der Therapie so früh wie möglich begonnen werden kann ohne die Sicherheit des Patienten zu gefährden. Die medizinische Betreuung ist unabhängig von der Studie bei beiden Patientengruppen gleich und somit gesichert.

Aufgrund der sehr komplexen Krankheitsvorgänge des Multiorganversagens, sowie der multifaktoriellen Entstehung der Critical Illness Polyneuropathie und Myopathie ist es nicht möglich die Intervention an gesunden Probanden durchzuführen. Ausserdem ist ein Ausdauer- und Krafttraining bei Gesunden und Sportlern schon gut erforscht. Jedoch sind die Intensitäten des Trainings nicht auf eine Intensivstation übertragbar.

Wie bereits oben beschrieben sind die Risiken gering. Mögliche Zwischenfälle sind zum Beispiel kurzandauernde Veränderungen von Blutdruck und Puls, das versehentliche Entfernen eines Schlauches oder ein Sturz bei der Mobilisation. Wie jedes Training, kann die Behandlung etwas anstrengend sein und zu Unannehmlichkeiten wie Muskelkater führen. Ansonsten sind bis jetzt keine weiteren Risiken oder Unannehmlichkeiten bekannt.

Das Studienthema ist klinisch relevant. Falls die physiotherapeutischen Massnahmen der Interventionsgruppe die der Kontrollgruppe überlegen sein würden, wird die Implementierung der neuen Behandlungstechniken im klinischen Alltag relativ einfach sein. Somit könnten zukünftige kritische Kranke mit einer höheren Leistungsfähigkeit und Selbständigkeit im Alltag aus dem Spital entlassen werden.

11.3 Andere ethische Aspekte

Die Studienteilnahme ist freiwillig. Der Patient kann seine Einwilligung zu jeder Zeit zurückziehen. Seine Daten werden in diesem Fall nur mit seinem Einverständnis weiterverwendet.
Die Kontrollgruppe erhält die bisher übliche Therapie und hat dementsprechend keinen Nachteil bezüglich der therapeutischen Behandlung.

12. Qualitätskontrolle und Qualitätssicherung: Beschreibung der Massnahmen

12.1 Gewährleistung des direkten Zugangs zu den Originaldaten, Erlauben von Audits, und Inspektionen durch die Behörden, und Ethikkommissionen, Durchführung des Monitorings

Der direkte Zugang zu den Originaldaten ist gewährleistet. Audits und Inspektionen durch die Behörden und Ethikkommission erlaubt.
Die Daten werden auf der Intensivstation mittel PDMS (Patient Data Management System) gesammelt. Dieses ist auf der Intensivstation durch bestehende Dokumentationspflicht (alle erhobenen Daten werden routinemässig ins PDMS eingegeben) gut implementiert. . Zudem kontrollieren die ärztlichen Betriebsleiter die Patientendaten regelmässig auf ihre Korrektheit. (z.B. APACHE II)
Ab Austritt Intensivstation werden die Messungen durch die verblindeten Assessoren auf Papier gesammelt, auf ihre Vollständigkeit überprüft und anschliessend durch Sabrina Eggmann in eine Excel Tabelle (Case Report Form) übertragen. In der Patientenakte wird lediglich ein Vermerk zur Studie notiert, um rechtzeitig über einen Spitalaustritt informiert zu werden.

12.2 Umgang mit Daten und Proben (Datenschutz), Archivierung (Ort, Dauer) und Vernichtung

Es werden keine Proben erhoben. Die gesammelten Daten werden nur verschlüsselt in die Case Report Forms eingetragen.
Die Krankenakten werden wie üblich archiviert.
Die Papierdaten werden nach Studienablauf durch einen Aktenvernichter entsorgt.

12.3 Direkt in den Case Report Forms einzutragende Daten (nicht in Krankenakten vermerkt) beschreiben

- Muskelkrafttests:
  - Voraussetzung: vorher 5 Befehle ausführbar:
    - Augen öffnen / schliessen → ja / nein
    - Tester anschauen → ja / nein
    - Mund öffnen und Zunge rausstrecken → ja / nein
    - mit dem Kopf nicken → ja / nein
    - Augenbrauen hochziehen, nachdem Tester auf 5 gezählt hat → ja / nein
  - Resultate der 6 Muskelgruppen nach Medical Research Council sum score (MRC):
    - Schulterabduktoren links → Zahl 0 – 5
    - Schulterabduktoren rechts → Zahl 0 – 5
    - Ellbogenflexoren links: → Zahl 0 – 5
    - Ellbogenflexoren rechts → Zahl 0 -5
    - Dorsalextensoren des Handgelenkes links → Zahl 0 – 5
    - Dorsalextensoren des Handgelenkes rechts → Zahl 0 - 5
    - Hüftgelenksflexoren links → Zahl 0 – 5

        - Hüftgelenksflexoren rechts → Zahl 0 – 5
        - Kniegelenksextensoren links → Zahl 0 – 5
        - Kniegelenksextensoren rechts → Zahl 0 – 5
        - Dorsalextensoren des Fussgelenkes links → Zahl 0 – 5
        - Dorsalextensoren des Fussgelenkes rechts → Zahl 0 – 5
    - Handkraft mit JAMAR Dynamometer ab M3 Ellbogenflexoren und Handgelenksdorsalextensoren:
        - rechts → in kg
        - links → in kg
    - Knieextensorenkraft mit Kraftmessgerät ab M3 Kniegelenksextensoren
        - rechts → in kg oder Newton
        - links → in kg oder Newton
- Bewegungsausmass, nur falls eingeschränkt::
    - Schultergelenk links: Abduktion / Flexion → in °
    - Schultergelenk rechts: Abduktion / Flexion → in °
    - Ellbogengelenk links: Flexion → in °
    - Ellbogengelenk links: Extension → in °
    - Ellbogengelenk rechts: Flexion → in °
    - Ellbogengelenk rechts: Extension → in °
    - Faustschluss links → in cm
    - Faustschluss rechts → in cm
    - Hüftgelenk links: Flexion → in °
    - Hüftgelenk rechts: Flexion → in °
    - Kniegelenk links: Flexion → in °
    - Kniegelenk links: Extension → in °
    - Kniegelenk rechts: Flexion → in °
    - Kniegelenk rechts: Extension → in °
    - Fussgelenk (OSG) links: Dorsalextension → in °
    - Fussgelenk (OSG) rechts: Dorsalextension → in °
- Functional Independence Measure (FIM)
    - Selbstversorgung
        - Essen/Trinken → Zahl 1 – 7
        - Körperpflege → Zahl 1 – 7
        - Baden/Duschen/Waschen → Zahl 1 – 7
        - Ankleiden oben → Zahl 1 – 7
        - Ankleiden unten → Zahl 1 – 7
        - Intimhygiene → Zahl 1 – 7
    - Kontinenz
        - Blasenkontrolle → Zahl 1 – 7
        - Darmkontrolle → Zahl 1 – 7
    - Transfers
        - Bett/Stuhl/Rollstuhl → Zahl 1 – 7
        - Toilettensitz → Zahl 1 – 7
        - Dusche/Badewanne → Zahl 1 – 7
    - Fortbewegung
        - Gehen (G)/Rollstuhl (R) → Zahl 1 – 7
        - Treppensteigen → Zahl 1 – 7
    - Kommunikation

        - Verstehen akustisch (A)/ Verstehen visuell (V) → Zahl 1 – 7
        - Ausdruck verbal (V)/ Ausdruck nonverbal (N) → Zahl 1 – 7
    - Kognitive Fähigkeiten
        - Soziales Verhalten → Zahl 1 – 7
        - Problemlösungsfähigkeit → Zahl 1 – 7
        - Gedächtnis → Zahl 1 – 7

- 6-Minuten-Gehtest → Distanz in m

- Timed-Up-And-Go Gehtest → Zeit in s

- Short Form 36 (SF 36) Fragebogen → mit vorgesehenem Auswertungsprogramm

13. Literatur

1. Ali, NA., O'Brien, JM., Hoffmann, SP., Phillips, G., Garland, A., Finley, JCW. et al. (2008). Acquired weakness, handgrip strength and mortality in critically ill patients. *Am J Respir Crit Care Med, 178,* 261–268.
2. American Thoracic Society. (2002). ATS Statement: Guidelines for the Six-Minute Walking Test. *Am J Respir Crit Care Med., 166*(1), 111-7.
3. Baldwin, CE., Paratz, JD. & Bersten, AD. (2012). Muscle strength assessment in critically ill patients with handheld dynamometry: An investigation of reliability, minimal detectable change, and time to peak force generation*. J Crit Care, in press.*
4. Burtin, C., Clerckx, B., Robbeets, C., Ferdinande, P., Langer, D., Troosters, T. et al. (2009). Early exercise in critically ill patients enhances short-term functional recovery. *Crit Care Med*, *37*(9), 2499-2505.
5. Büsching, G., Hilfiker, R., Mangold, F., Messmer, G., van Oort, E., Schädler, S. et al. (2009). *Assessments in der Rehabilitation: Band 3: Kardiologie und Pneumologie* (1. Auflage). Bern: Verlag Hans Huber.
6. Debrunner, HU. (1971). Gelenksmessung (Neutral-0-Methode) Längenmessung Umfangmessung.
7. Doherty, N. & Steen, CD. (2010). Critical illness polyneuromyopathy (CIPNM); rehabilitation during critical illness. Therapeutic options in nursing to promote recovery. A review of the literature*. Intensive Crit Care Nurs. 26*(6), 353-362.
8. De Jonghe, B., Bastuji-Garin, S., Sharshar, T., Outin, H. & Brochard,L. (2004). Does ICU-acquired paresis lengthen weaning from mechanical ventilation? *Intensive Care Med., 30*(6),1117-21.
9. Elliott, D., Denehy, L., Berney, S. & Alison, JA. (2011). Assessing physical function and activity for survivors of a critical illness: A review of instruments. *Aust Crit Care, 24*,155-166.
10. Fan, E., Zanni, JM., Dennison, CR., Lepre, SJ. & Needham, DM. (2009). Critical Illness Neuromyopathy and Muscle Weakness in Patients in the Intensive Care Unit. *AACN Adv Crit Care, 20*(3), 243-53.
11. Fan, E. (2010). What is stopping us from *early* mobility in the intensive care unit? *Crit Care Med, 38*(11), 2254-55.
12. Garzon-Serrano, J., Ryan, C., Waak, K., Hirschberg, R., Tully, S., Bittner, EA. et al. (2012). Early Mobilization in Critically Ill Patients: Patients' Mobilization Level Depends on Health Care Provider's Profession. *PM R. 3*, 307-313.
13. Gosselink, R., Bott, J., Johnson, M., Dean, E., Nava, S., Norrenberg, M. et al. (2008). Physiotherapy for adult patients with critical illness: recommendations of the European Respiratory Society and European Society of Intensive Care Medicine Task Force on Physiotherapy for Critically Ill Patients*. Intensive Care Med, 34*(7):1188-99.
14. Hermans, G., Clerckx, B., Vanhullebusch, T., Segers, J., Vanpee, G., Robbeets, C. et al. (2012). Interobserver agreement of medical research council sum-score and handgrip strength in the intensive care unit. *Muscle Nerv, 45*(1), 18-25.
15. Hofhuis, JGM., Spronk, PE., van Stel, HF., Schrijvers, GJP., Rommes, JH.& Bakker, J. (2008). The Impact of Critical Illness on Perceived Health-Related Quality of Life During ICU Treatment, Hospital Stay, and After Hospital Discharge. *Chest, 133*(2), 377-85.
16. Judemann, K., Lunz, D., Zausig, YA., Graf, BM. & Zink, W. (2011). Erworbene Muskelschwäche beim kritisch Kranken: Critical-Illness-Polyneuropathie und Critical-Illness-Myopathie. *Anaesthesist, 60*, 887-901.
17. Kress, JP. (2009). Clinical trials of early mobilization of critically ill patients. *Crit Care Med, 37*(10), 442-47.
18. Morris, PE., Goad, A., Thompson, C., Taylor, K., Harry, B., Passmore, L. et al. (2008). Early intensive care unit mobility therapy in the treatment of acute respiratory failure. *Crit Care Med, 36*(8), 2238-43.
19. National Institute for Health and Clinical Excellence. (2009). Critical Illness Rehabilitation Guidelines. NICE clinical guideline 83.
20. Oesch, P., Hilfiker, R., Keller, S., Kool, J., Schädler, S., Tal-Akabi, A., et al. (2007). *Assessments in der muskuloskelettalen Rehabilitation* (1. Auflage). Bern: Verlag Hans Huber.

21. Pohlman, MC., Schweickert, WD., Pohlman. AS., Nigos, C., Pawlik, AJ., Esbrook, CL. et al. (2010). Feasibility of physical and occupational therapy beginning from initiation of mechanical ventilation*. Crit Care Med, 38*(11), 2089-94.
22. Radlinger, L., Bachmann, W., Homburg, J., Leuenberger, U. & Thaddey, G. (1998). *Rehabilitatives Krafttraining.* Stuttgart: Georg Thieme Verlag.
23. Redelmeier, DA., Bayoumi, AM., Goldstein, RS., Guyatt, GH. (1997). Interpreting Small Differences in Functional Status: The Six Minute Walk Test In Chronic Lung Disease Patients. *Am J Respir Crit Care Med,. 155*(4), 1278-82.
24. Schädler, S., Kool, J., Lüthi, H., Marks, D., Oesch, P., Pfeffer. A. & Wirz, A. (2006*). Assessments in der Neurorehabilitation.* (1. Auflage). Bern: Verlag Hans Huber.
25. Schweickert, W.D., Pohlmann, M.C., Nigos, C., Pawlik, A.J., Esbrook, C.L., Spears, L. et al. (2009). Early physical and occupational therapy in mechanically ventilated, critically ill patients: a randomised controlled trial. *Lancet, 373*, 1874-1826.
26. Stevens, RD., Marshall, SA., Cornblath, DR., Hoke, A., Needham, DM., de Jonghe, B. et al. (2009). A framework for diagnosing and classifying intensive care unit-acquired weakness. *Crit Care Med, 37*(10), 299-308.
27. Stockley, RC., Morrison, J., Rooney, J. & Hughes, J. (2012). Move it or lose it?: A survey of the aims of treatment when using passive movements in intensive care. *Intensive Crit Care Nurs., 28*(2), 82-87.
28. Truong, AD., Fan, E., Brower, RG. & Needham, DM. (2009). Bench-to-bedside review: Mobilizing patients in the intensive care unit – from pathophysiology to clinical trials. *Crit Care,13*(4), 216.
29. Van der Schaaf, M., Beelen, A., Dongelmans, DA., Vroom, MB. & Nollet, F. (2009). Functional stuatus after intensive care: a challenge for rehabilitation professionals to improve outcome. *J Rehabil Med, 41,* 360–366.
30. Winkelman, C., Johnson, KD., Hejal, R., Gordon, NH., Rowbottom, J., Daly, J. et al. (2012). Examining the positive effects of exercise in intubated adults in ICU: A prospective repeated measures clinical study. *Intensive Crit Care Nurs,* in press.
31. Zeppos, L., Patman, S., Berney, S., Adsett, JA., Bridson, JM. & Paratz, JD. (2007). Physiotherapy intervention in intensive care is safe: an observational study. *Aust J Physiother., 53*(4), 279-83.

Translated study protocol

Translated with www.DeepL.com/Translator

Effects of early, combined endurance and resistance training in mechanically ventilated, critically ill patients: a randomised controlled trial.

1. Title of the study protocol

1.1 Title: Effects of early, combined endurance and resistance training in mechanically ventilated, critically ill patients: a randomised controlled trial.

1.1.1 Version 3 of 17th September 2012 (CEC-BE: 122/12, DLF Study: 2221, UTN (University Trial Number): U1111-1134-4362, Registration No.: DRKS00004347)

1.1.2 Signature of examiner Prof. Dr. med. Stephan Jakob: Bern, 17.9.12

3. Objectives and purpose

3.1 Background, justification and objective of the study

In the intensive care unit (ICU) with the main focus on resuscitation and survival, traditionally little early attention is paid to neuromuscular functions. The critical disease, often sepsis and multiorgan failure, is in the foreground. In the 1980s, Bolton et al. described unexplained prolonged weaning of ventilation, reduced muscle reflexes and generalized muscle weakness in patients with sepsis. They called this phenomenon critical illness polyneuropathy. [Bolton, 1986]

Today there are clear definitions and diagnostic criteria for this phenomenon. The "acquired skeletal muscle weakness in critically ill patients" („ICU-acquired weakness", ICUAW) describes a clinically diagnosed, newly occurring diffuse muscle weakness in intensive care patients without a definable cause, except for the critical illness itself and a Medical Research Council summary score of < 48 [Stevens, 2009].

Acquired skeletal muscle weakness in critically ill patients in intensive care is associated with poor outcomes. On the one hand, ICUAW increases the ventilation time up to 2.5 times. [De Jonghe, 2004] More than 30% of survivors continue to suffer from poor functional status one year after intensive care. Patients are particularly restricted in their social behaviour and mobility. The quality of life is severely impaired. [Van der Schaaf, 2009]

Even bed rest and immobilization alone can cause serious complications, for example skeletal muscle atrophy and weakness, joint contractures, atelectases, decubitus, thrombosis, systemic inflammation, etc. Countermeasures include resistance exercises, passive stretching exercises and mobilisation. [Brower, 2009]

Physiotherapists with their specific knowledge of neurological and musculoskeletal functions could play an important role in the intensive care environment. Their expertise often enables them to achieve higher levels of mobility than other health professions. [Garzon-Serrano, 2012]. In addition, new studies show that early mobilisation and physiotherapy in the intensive care unit is not only safe and feasible, but also associated with a better hospital outcome. [Morris, 2008, Burtin,2009, Schweickert, 2009]. Early mobilisation significantly shortened the stay in the intensive care unit and hospital. [Morris, 2008]. Early bed-cycle training improved performance (measured with the 6 min walk test) and quality of life (measured with the questionnaire SF-36). Burtin, 2009] Further, patients with physiotherapy

reached a higher level of daily activities and spent fewer days with delirium. [Schweickert, 2009]
However, according to the European Respiratory Society and European Society of Intensive Care Medicine Task Force, there is only evidence of Level C-D for physiotherapy in intensive care units. [Gosselink, 2008]. Also missing is a general agreement or an evidence-based guideline on intensity, frequency, optimal start and duration of therapy. [NICE guidelines, 2009] The evidence for the treatment of joint mobility in sedated patients is very unclear. Stockley, 2012] Furthermore, most of the studies mentioned above were conducted in a respiratory or medical intensive care unit. [Morris, 2008, Schweickert, 2009, Pohlman, 2010] Accordingly, data with surgical or neurological patients are missing in intensive care. Further randomised controlled trials are therefore necessary to evaluate possible short- and long-term benefits for the muscle strength, physical functions and quality of life of patients and to determine the usefulness and effectiveness of interventions, e.g. mobilization aids or bicycle ergometers. [Fan, 2009]
Accordingly, the aim of this study in an interdisciplinary intensive care unit is to combine an early mobilization concept with endurance and strength training.
First and foremost, the feasibility and safety with a mixed patient population should be determined and, as a primary outcome, the performance and independence in everyday life should be measured. As a secondary outcome, strength, mobility, and quality of life are measured. Variables for treatment safety are oxygen consumption, oxygenation and hemodynamics during treatment.
Through the planned study, we expect new knowledge on the safety and implementation of early physiotherapy in the intensive care unit and a knowledge gain on its short-term benefits, in addition to performance, above all the effect on mobility and strength, which are important prerequisites for carrying out everyday activities. In addition, the oxygen consumption in rest and exertion can help us to improve our health.

3.2 Questions, study population

3.2.1 Main question:

Do patients achieve higher performance and independence in everyday life when leaving hospital with appropriate endurance and strength training in an interdisciplinary intensive care unit?

3.2.2 Further questions:

- Is early mobilisation with appropriate endurance and strength training in an interdisciplinary intensive care unit safe and feasible?
- Can early mobilisation and training improve strength and mobility as soon as the patient leaves the intensive care unit?
- Is there a direct connection between the components mobility and strength and independence in everyday life?
- Is there a connection between the outcome data and the complications of bed rest (weakness / ICUAW, contracture, atelectasis and decubitus, pneumonia, thrombosis and delir)?
- Do patients in the treatment group have a better quality of life 6 months after leaving hospital?

3.2.3 Study population:
Inpatients of the Department of Intensive Care Medicine who have an expected time of mechanical ventilation of at least 72 hours in the intensive care unit will be examined. Since muscle loss already begins in the first days of bed rest, physiotherapeutic intervention should begin as early as possible. Therefore, the aim is to detect and treat patients at an early stage who are at risk of bed rest complications. Accordingly, inclusion was chosen with an expected ventilation time of at least 72 hours.

3.3 Hypotheses
- Early mobilisation combined with appropriate endurance and strength training in an interdisciplinary intensive care unit is safe and feasible. This improves the performance of these patients, which leads to greater independence in everyday life.
- Early mobilisation combined with appropriate endurance and strength training in an interdisciplinary intensive care unit can improve strength and mobility. The quality of life after 6 months is improved by the early training.

4. Study design
4.1 Main target parameters, and secondary target parameters
- The main target parameters are performance and independence in everyday life when leaving hospital.
- Secondary target parameters are strength, mobility and independence in everyday life when leaving the intensive care unit, as well as the quality of life 6 months after leaving the hospital.
- Further target parameters: Number of days in intensive care and in hospital

4.2 Study design
- Randomised controlled trial, prospective with follow-up after 6 months
- Main study (Phase 2 study, i.e. pilot RCT with focus on feasibility and preliminary effectiveness - interim results will be published for Sabrina Eggmann's MSc thesis at Danube University Krems, course: Master of Science in Neurorehabilitation)
- Intervention study with blinding of the statistician and assessor
- Monocentric
- National

4.2.1 Tabular overview of examinations with time grid (flowchart)

Examination	Timepoint
**Progress reviews**	Daily during the intervention
**Security checks**	Daily during the intervention
**Strength measurements**	Days 7, 21, 35,... discharge intensive care unit
**Functional Independence Measure**	Day 7, 21, 35,... Departure intensive care unit / hospital
**Mobility measurement**	Day 7 and exit intensive care unit
**6-minute walk test**	hospital discharge
**Timed-Up-And-Go**	hospital discharge
**Questionnaire SF-36**	6 months after leaving hospital

4.2.2 Course of studies

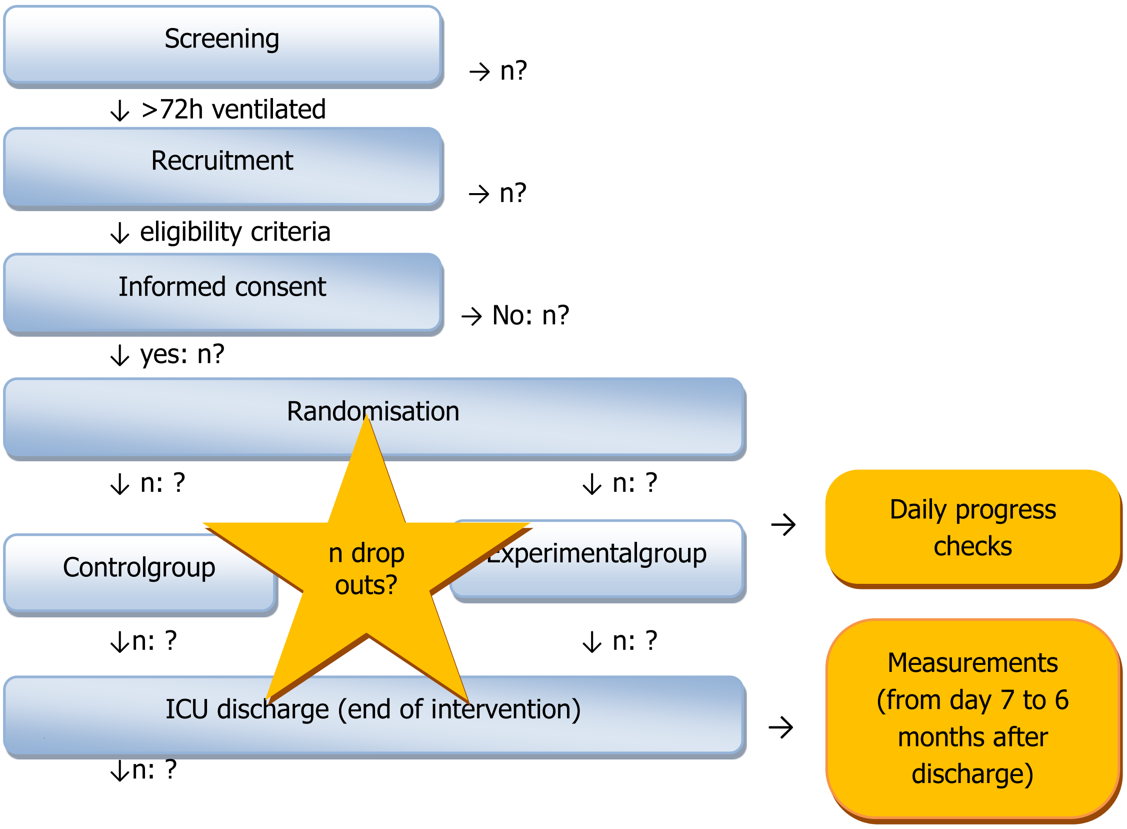

4.2.3 Interventions

4.2.3.1 Control Group

- Start of physiotherapy as soon as the attending physician prescribes, daily screening by physiotherapist (PT)
- Breathing or swallowing therapy as required
- Physiotherapy 5-7 times a week, decision by treating PT, according to existing protocol
- Physiotherapy 1x daily (with additional swallowing therapy, 2x daily)
- Sedated patients: passive movement therapy with 3-10 repetitions (Whg) of each joint, in all possible directions of the respective patient position, positioning according to the existing decubitus protocol
- Awake patients: passive, active-assisted or active movement therapy, depending on the patient's possibility (decision by treating PT) and positioning
- Mobilisation starting at the edge of the bed, as prescribed by the attending physician

4.2.3.2 Intervention group

General information:

- Start of physiotherapy as soon as the attending physician prescribes, daily clarification by PT
- Breathing or swallowing therapy as required
- Physiotherapy 5-7 times a week, decision by treating PT, according to existing protocol

– Physiotherapy 1x daily (Level I-II), 1-2x daily (Level III), 2x daily (Level IV-VI), max. 2x daily (including swallowing therapy)
– Mobilisation starting in bed, on prescription of the attending physician
– if possible reduction of sedatives for therapy (after prescription of the treating physician: level II)

Therapy overview:

Level I	Level II	Level III	Level IV	Level V	Level VI
sedated	Sedation-stopps	No contra-indications	10min edge of bed	50% help transfer, M3 4ceps	50% help stand
p ROM	p / a.ass / a ROM	p / a.ass / a ROM	p / a.ass / a ROM	a.ass / a ROM	a.ass / a ROM
Positioning	In-bed	Edge-of-bed	Chair	Stand	Walk
MOTOmed Letto 2 (bed cycling) Training					

p = passive, a.ass = active - assisted, a = active, ROM = range of motion therapy

Exercise therapy and strength training:
– pROM: with 3-10 Whg, each joint in all possible directions of the respective patient position, if possible including everyday activities (ADL).
– a.ass. ROM: with as much support as necessary to achieve full range of motion, 3-10 Whg, each joint in all possible directions of the respective position, if possible including ADL.
– specific mobility exercises for hands and feet as a home program a corresponding sheet hangs by the patient's bed.
– a ROM / Strength training: as soon as a.ass. ROM with < 25% help the exercises are performed with weight (start at 450g, 1kg, 2kg, etc.) or therapeutic resistance. The therapeutic guidance aid serves above all to achieve the full extent of movement and the qualitatively correct execution.
– Muscle groups for strength training: hip joint abductors, knee joint extensors, foot dorsal extensors, shoulder joint flexors and rotators, elbow flexors (additional individual training according to PT decision, e.g. trunk muscles, hip joint extensors)
– Training is conducted at an average load intensity (50-70%) with 8-12 Whg and 2-5 series, with a break of at least 2 minutes between series.
– Monday, Wednesday and Friday: strength training for upper extremity and correspondingly Tuesday and Thursday: lower extremity
– As soon as a patient performs the strength exercises correctly, he may practice them with relatives or care during the day. In this case, the exercise program is hung near the patient's bed.

Endurance training with MOTOmed Letto2, bed bike ergometer:
– Intensity: 1x daily, 5x weekly
– Starting position of the patient: supine position with 30° elevated headrest
– sedated patients: passive, up to 20 min and with maximum 20 rpm

- mixed active - passive pedalling patients: up to 20 min, 20 rpm, 0 degree resistance
- 20 min active patients (grade 0): increase of resistance
- 20 min active patients (grade 2): increase to 30 min, then resistance increase by 1 degree every 2nd day, up to grade 6
- 30 min active patients (grade 6): increase of time up to max. 60min
- individual adjustments / increases possible (PT decision)

Mobilisation with ADL involvement:
- Level I: sedated patients: Positioning according to decubitus protocol
- Level II: Sedation stops: Upright U-position in bed
- Level III: bed edge, individual positioning (e.g. with backrests, armrests, etc.)
- Level IV: Armchair, individual transfer (e.g. deep with sheet, deep without sheet, high via stand → PT decision), prerequisite: min. 10min bed edge possible
- Level V: Stand (with auxiliary person → PT / nurse), requirements: force of M3 in the M. Quadriceps, < 50% transfer support
- Level VI: walking (with aids or ancillary person → PT / nurse), prerequisites: <50% standing support, steps can be taken on site
- ADL is individually adjusted and included, decision by treating PT (e.g. turning in bed, bridging, balance in sit/stand, sit-to-stand, transfer, etc.)

4.2.4 Study duration per patient
The intervention takes place during the intensive care stay, therefore the duration of the study is individual for each patient. The last measurement takes place 6 months after leaving the hospital in the form of a questionnaire.

4.2.5 Abort criteria
Large fluctuations in pulse, blood pressure or oxygen saturation during treatment, or a strong increase in oxygen consumption can lead to a study abortion. The decision to abort lies with the study physician.
A therapy session can be terminated by the therapist (see defined termination criteria 7.1.1). In this case, the therapy is not resumed until the next day.

4.2.6 Conditions for unblinding
The patient and the treatment team are not blinded.

4.3 Measures to minimize bias
- Randomization
- Blinding statisticians and assessors
- The medical treatment remains the same for both groups

5. Selection of test subjects
5.1 Recruitment
Recruitment of study participants: Screening by the intensive care unit research team using the Patient Data Management System (PDMS)

Willingness to participate: Deferred consent (within 72 hours as recommended by the AGEK), present presumed expression of will by family members or a signed declaration of consent by the patient, if possible, obtained by the attending physician.

5.2 Inclusion criteria
- ≥ 18 years
- expected ventilation of > 72h in interdisciplinary intensive care unit
- qualitatively independent before illness

5.3 Exclusion criteria
- Contraindication for MOTOmed bed bike ergometer (unstable fractures/ open wounds in the upper and lower extremities/ pelvis/ abdomen)
- Weight > 150kg
- Existing neuromuscular / neurological disease that led to permanent limitations in strength, mobility, performance or independence in everyday life
- previous hospital stay of more than 10 days
- Therapy limitation due to expected poor outcome
- Inclusion in other intervention study
- Admission diagnoses that prevent a walking test after acute stage: e.g. patients with pronounced cerebral vascular insult, severe craniocerebral trauma, spinal cord injury with tetra- or paraplegia, etc.

6. Evaluation of effectiveness

6.1 Effectiveness parameters: Measuring methods and times

6.1.1 Main target parameters
- Performance: 6-minute walking test (6MWT) and Timed-Up-And-Go (TUG) at hospital discharge, measured by physiotherapists (PT)
- Independence in everyday life: Functional Independence Measure (FIM) on discharge from the intensive care unit and hospital, completed by the treatment team (nursing and PT)
- Milestone achievement during the intensive care stay in number of days for Mobilisation level (bed edge / armchair / stand / walk)

6.1.2 Secondary target parameters
- Strength, tests from day 7 (progress checks every 14d) and in case of discharge from the intensive care unit, by physiotherapists, prerequisite: patient follows 3 of 5 commands:
- Medical Research Council sum score (MRC) for 6 muscle groups
- Grip strength by means of JAMAR dynamometer
- Quantitative muscle test for knee joint extensors using a force measuring device (hand held dynamometer)
- Range of motion (ROM) with goniometer according to neutral-zero method, tests on day 7 and in intensive care units by physiotherapists
- Quality of life: Short Form 36 Health questionnaire (SF-36) 6 months after leaving hospital
- Number of days in intensive care and in hospital

7. Safety assessment

7.1 Safety parameters: Measuring methods and times

- Continuous 24h monitoring of oxygen saturation, pulse and blood pressure according to intensive care unit standard, data acquisition in the patient management system (PDMS)
- Oxygen consumption (VO2): indirect calorimetry 30min before, during and 15 minutes after treatment of ventilated patients, performed by the treating intensive care specialist, data acquisition with PDMS
- Daily recording of the following complications: ICUAW, atelectasis, pneumonia, decubitus, contracture, thrombosis, delirium and critical illness polyneuropathy or myopathy
- Recording the number of treatments performed

7.1.1 defined criteria for interruption or termination of therapy:

- subjective severe fatigue / exertion of the patient
- medically prescribed limits undercut / limits exceeded (especially haemodynamics, oxygenation and oxygen consumption)
- ventilator asynchrony
- no patient cooperation
- severe pain
- adverse event

7.1.2 Progress checks:

Findings before, during and after treatment (if ascertainable):

- Richmond Agitation Sedation Scale (RASS)
- Pain on Visual Analog Scale (VAS)
- Stress perception - BORG scale

Special findings during treatment for progress documentation:

- extent of movement
- strength training parameters
- endurance training parameters
- Mobilisation and ADL parameters

7.2 Ensuring follow-up of subjects after adverse events

Continuous 24h monitoring and safety analysis by PDMS (Patient Data Management System)
Listing of all harmful events during and up to 15 minutes after treatment that occur again and do not respond to intervention or persist despite therapy interruption:

- new haemodynamically relevant arrhythmia
- new oxygenation disturbance (SpO2 < 85%)
- new unstable haemodynamics
- other damaging events:
- accidental removal of a tube, catheter or the like
- fall
- other injury

serious adverse event: clinical assessment of the investigator as to whether it is relevant to the study

8. Statistics

8.1 Definition of the primary endpoint and secondary endpoints
The minimal clinically important difference in the 6-minute walk test for patients with chronic lung disease was validated at 50m. [Redelmeier, 1997, ATS, 2002].
The following basic patient data are collected for group comparison:

- Age, gender, weight, body mass index (BMI)
- APACHE II (Acute Physiology and Chronic Health Evaluation II)
- SOFA score (Sequential Organ Failure Assessment score)
- TISS (Therapeutic Intervention Scoring System) first entry and last entry before leaving intensive care unit
- Medication, number of days and daily dose:
    - Relaxants
    - vasopressors
    - sedatives
    - Opiates
    - steroids
    - insulin
- laboratory values : CRP (C-Reactive Protein), creatinine, bilirubin, thrombocytes (Tc), leukocytes (Lc), hemoglobin (Hb) existing values at entry in intensive care unit, after study inclusion and at exit from intensive care unit
- Admission diagnosis and diagnostic group
- the presence of a tracheotomy or respiratory tube

8.2 Planned number of test persons with comprehensible reason (possibly power analysis)
To demonstrate a difference of 50m in a 6-minute walk test with a statistical power of 80% and a α of 0.05 a sample size of 36 patients per group is required. With an expected drop-out of 20% and an expected high correlation of the two primary endpoints (6-minute walk test and FIM), 115 patients will be included in the study. In the year the Inselspital Bern has an average of 90 patients who are ventilated for more than 3 days and meet the inclusion criteria. Based on previous studies [Winkelman, 2012] an inclusion of approx. 74% is expected (no invasive methods, therapy is usually well received.) Accordingly, a study duration of 19 months is planned: September 17, 2012 - May 31, 2014.

8.3 Description of the planned statistical methods and the planned interim evaluations
Evaluations with the SPSS program:

- Descriptive statistics regarding the content
- Comparison within and between the groups
- Comparison of demographic data
- significant differences

For group comparison: students-t-test (normal distribution) or Mann-Withney-U test (no normal distribution)
For comparison of proportions between the two groups: Fisher's exact test.

For repetitive data: ANOVA Analysis

Interim evaluation of the data for Sabrina Eggmann's master thesis in September 2013.

8.4 Planned significance level
statistical power of 80% and an α of 0.05

8.5 Dealing with missing data and data in the event of premature discontinuation of studies by participants

- Analysis using intention-to-treat and protocol
- If the study is terminated prematurely, the patient is asked whether the data may continue to be used. Otherwise, they will be destroyed.

8.6 Definition of the evaluation groups (Intention To Treat, Per Protocol, etc.)
Analysis using intention-to-treat and protocol

9 Study specific precautions and duties
Patients have no duties and no precautions to take.

10. Duties of the auditor
10.1 The study will be conducted in accordance with the Protocol, GCP, and applicable regulatory requirements.
10.2 Serious adverse events, (protocol) changes will be reported. Interim and final reports are sent to the Ethics Committee.
10.3 Statement on cover for damages
Guaranteed coverage by the Inselspital, University Hospital Bern

11 Ethical considerations
11.1 Assessment of the risk-benefit ratio
Intensive care physiotherapy is safe. The incidence of harmful physiological events is very low at 0.2%. Zeppos, 2007] Harmful events are unusual; in 498 physiotherapy and occupational therapy sessions Schweickert et al. had an accidentally removed radial artery catheter and oxygen desaturation of 80%. [Schweickert, 2009] There were no harmful events at Morris et al. [Morris, 2008] The benefit to patients was high in both studies. Patients were in bed for less time, in hospital and in the intensive care unit at Morris et al. and left the hospital with a higher degree of independence in everyday life at Schweickert et al. An early start of physiotherapy already in the first phases of a critical illness improves functional and psychological endpoints in ventilated patients [Schweickert, 2009]. This is an essential point for the return to life and remains impaired in more than 30% of patients after a critical illness. van der Schaaf, 2009] The burden on survivors of a critical illness is generally very high. Accordingly, effective therapies are urgently needed and should start early. [Hofhuis, 2008]
Physiotherapy has long been an integral part of the intensive care unit at the Inselspital Bern. The treating therapists are experienced and there is a good interdisciplinary cooperation. Early mobilisation is well established and there are few incidents. The study is

supported and supported by Prof. Dr. med. Jukka Takala, Director and Chief Physician of the University Hospital for Intensive Care and Martin L. Verra, MPtSc. director of the Institute of Physiotherapy.

11.2 Description of why particularly vulnerable subjects are included
Since the aforementioned studies give us indications that therapy should start early and it is safe to start it in the intensive care unit, this particularly vulnerable patient group is selected. In order to include the patient's will as far as possible, his relatives are asked about his presumed will within 72 hours. In addition, a physician independent of the study will be consulted so that therapy can begin as early as possible without endangering the patient's safety. Medical care is the same for both patient groups, regardless of the study.

Due to the very complex disease processes of multiorgan failure, as well as the multifactorial development of critical illness polyneuropathy and myopathy, it is not possible to carry out the intervention on healthy subjects. In addition, endurance and strength training is already well researched in healthy people and athletes. However, the intensities of the training cannot be transferred to an intensive care unit.
As already described above, the risks are low. Possible incidents include short-lasting changes in blood pressure and pulse, accidental removal of a tube or a fall during mobilisation. Like any training, the treatment can be a bit strenuous and lead to inconveniences such as sore muscles. No other risks or inconveniences are known so far.
The study topic is clinically relevant. If the physiotherapeutic measures of the intervention group would be superior to those of the control group, the implementation of the new treatment techniques in everyday clinical practice will be relatively simple. Thus, future critical patients could be discharged with a higher performance and independence in everyday life from the hospital.

11.3 Other ethical aspects
Participation in the study is voluntary. The patient can withdraw his consent at any time. In this case his data will only be used with his consent.
The control group receives the usual therapy and therefore has no disadvantage in terms of therapeutic treatment.

12. Quality control and quality assurance: description of measures

12.1 Ensuring direct access to the original data, allowing audits and inspections by the authorities, and ethics committees, carrying out monitoring.
Direct access to the original data is guaranteed. Audits and inspections by the authorities and the ethics committee are permitted.
The data is collected in the intensive care unit using PDMS (Patient Data Management System). This is well implemented in the intensive care unit due to the existing documentation obligation (all collected data is routinely entered into the PDMS). In addition, the medical managers regularly check the patient data for correctness. (e.g. APACHE II)
After leaving the ICU, the blinded assessors collect the measurements on paper, check them for completeness and then transfer them to an Excel table (Case Report Form) by Sabrina

Eggmann. In the patient file, only a note on the study is noted in order to be informed in good time of a hospital discharge.

12.2 Handling data and samples (data protection), archiving (location, duration) and destruction
No samples are collected. The collected data is only entered into the Case Report Forms in encrypted form.
The medical records are archived as usual.
The paper data is disposed of after the course of study by a paper shredder.

12.3 Describe data to be entered directly in the Case Report Forms (not noted in medical records)

- Muscle strength tests:
  - Prerequisite: 5 commands can be executed beforehand:
    - open / close eyes yes / no
    - View tester yes / no
    - Open your mouth and stick out your tongue yes / no
    - nod your head yes / no
    - Raise eyebrows after tester counts down to 5 yes / no
- Results of the 6 muscle groups according to Medical Research Council sum score (MRC):
  - shoulder abductors left number 0 - 5
  - shoulder abductors right number 0 - 5
  - Elbow flexors left: Number 0 - 5
  - elbow flexors right number 0 -5
  - Dorsal extensors of the left wrist Number 0 - 5
  - Dorsal extensors of the right wrist Number 0 - 5
  - hip joint flexors left number 0 – 5
  - hip joint flexors right number 0 - 5
  - Knee joint extensors left Number 0 - 5
  - right knee joint extensors number 0 - 5
  - Dorsal extensors of the left ankle Number 0 - 5
  - Dorsal extensors of the right ankle Number 0 - 5
- Manual force with JAMAR dynamometer from M3 elbow flexors and wrist dorsal extensors:
  - right in kg
  - left in kg
- Knee extensor force with force gauge from M3 and higher Knee joint extensors
  - right in kg or Newton
  - left in kg or Newton
- Extent of movement, only if limited::
  - Shoulder joint left: Abduction / Flexion in °
  - Right shoulder joint: abduction / flexion in °.
  - Elbow joint left: Flexion in °
  - Elbow joint left: Extension in °
  - right elbow joint: flexion in °

- right elbow joint: extension in °
- fist closure left in cm
- fist closure right in cm
- Hip joint left: Flexion in °
- hip joint right: flexion in °
- Knee joint left: Flexion in °
- Knee joint left: Extension in °
- right knee joint: flexion in °
- Knee joint right: Extension in °
- Ankle joint (OSG) left: Dorsal extension in °
- Ankle joint (OSG) right: dorsal extension in °

- Functional Independence Measure (FIM)
  - Self-sufficiency
    - Food/drink → Number 1 - 7
    - Body care → number 1 - 7
    - bath/shower/wash → number 1 - 7
    - Dress up → number 1 - 7
    - Dressing down → number 1 - 7
    - Intimate hygiene → number 1 - 7
  - Continence
    - Bladder control → Number 1 - 7
    - bowel control → number 1 - 7
  - Transfers
    - bed/chair/wheelchair → number 1 - 7
    - toilet seat → number 1 - 7
    - shower/bathtub → number 1 - 7
  - locomotion
    - Walking (G)/Wheelchair (R) → Number 1 - 7
    - Climbing stairs → number 1 - 7
  - Communication
    - Understanding acoustic (A)/understanding visual (V) → Number 1 - 7
    - Expression verbal (V)/ expression nonverbal (N) → Number 1 - 7
  - cognitive abilities
    - Social behaviour → number 1 - 7
    - Problem solving ability → Number 1 - 7
    - memory → number 1 - 7

- 6-minute walk test → distance in m

- Timed-Up-And-Go walk test → time in s

- Short Form 36 (SF 36) questionnaire → with evaluation program provided

13. References

32. Ali, NA., O'Brien, JM., Hoffmann, SP., Phillips, G., Garland, A., Finley, JCW. et al. (2008). Acquired weakness, handgrip strength and mortality in critically ill patients. *Am J Respir Crit Care Med, 178,* 261–268.

33. American Thoracic Society. (2002). ATS Statement: Guidelines for the Six-Minute Walking Test. *Am J Respir Crit Care Med., 166*(1), 111-7.
34. Baldwin, CE., Paratz, JD. & Bersten, AD. (2012). Muscle strength assessment in critically ill patients with handheld dynamometry: An investigation of reliability, minimal detectable change, and time to peak force generation*. J Crit Care, in press.*
35. Burtin, C., Clerckx, B., Robbeets, C., Ferdinande, P., Langer, D., Troosters, T. et al. (2009). Early exercise in critically ill patients enhances short-term functional recovery. *Crit Care Med*, *37*(9), 2499-2505.
36. Büsching, G., Hilfiker, R., Mangold, F., Messmer, G., van Oort, E., Schädler, S. et al. (2009). *Assessments in der Rehabilitation: Band 3: Kardiologie und Pneumologie* (1. Auflage). Bern: Verlag Hans Huber.
37. Debrunner, HU. (1971). Gelenksmessung (Neutral-0-Methode) Längenmessung Umfangmessung.
38. Doherty, N. & Steen, CD. (2010). Critical illness polyneuromyopathy (CIPNM); rehabilitation during critical illness. Therapeutic options in nursing to promote recovery. A review of the literature*. Intensive Crit Care Nurs. 26*(6), 353-362.
39. De Jonghe, B., Bastuji-Garin, S., Sharshar, T., Outin, H. & Brochard,L. (2004). Does ICU-acquired paresis lengthen weaning from mechanical ventilation? *Intensive Care Med., 30*(6),1117-21.
40. Elliott, D., Denehy, L., Berney, S. & Alison, JA. (2011). Assessing physical function and activity for survivors of a critical illness: A review of instruments. *Aust Crit Care, 24*,155-166.
41. Fan, E., Zanni, JM., Dennison, CR., Lepre, SJ. & Needham, DM. (2009). Critical Illness Neuromyopathy and Muscle Weakness in Patients in the Intensive Care Unit. *AACN Adv Crit Care, 20*(3), 243-53.
42. Fan, E. (2010). What is stopping us from *early* mobility in the intensive care unit? *Crit Care Med, 38*(11), 2254-55.
43. Garzon-Serrano, J., Ryan, C., Waak, K., Hirschberg, R., Tully, S., Bittner, EA. et al. (2012). Early Mobilization in Critically Ill Patients: Patients' Mobilization Level Depends on Health Care Provider's Profession. *PM R. 3*, 307-313.
44. Gosselink, R., Bott, J., Johnson, M., Dean, E., Nava, S., Norrenberg, M. et al. (2008). Physiotherapy for adult patients with critical illness: recommendations of the European Respiratory Society and European Society of Intensive Care Medicine Task Force on Physiotherapy for Critically Ill Patients*. Intensive Care Med, 34*(7):1188-99.
45. Hermans, G., Clerckx, B., Vanhullebusch, T., Segers, J., Vanpee, G., Robbeets, C. et al. (2012). Interobserver agreement of medical research council sum-score and handgrip strength in the intensive care unit. *Muscle Nerv, 45*(1), 18-25.
46. Hofhuis, JGM., Spronk, PE., van Stel, HF., Schrijvers, GJP., Rommes, JH.& Bakker, J. (2008). The Impact of Critical Illness on Perceived Health-Related Quality of Life During ICU Treatment, Hospital Stay, and After Hospital Discharge. *Chest, 133*(2), 377-85.
47. Judemann, K., Lunz, D., Zausig, YA., Graf, BM. & Zink, W. (2011). Erworbene Muskelschwäche beim kritisch Kranken: Critical-Illness-Polyneuropathie und Critical-Illness-Myopathie. *Anaesthesist, 60*, 887-901.
48. Kress, JP. (2009). Clinical trials of early mobilization of critically ill patients. *Crit Care Med, 37*(10), 442-47.
49. Morris, PE., Goad, A., Thompson, C., Taylor, K., Harry, B., Passmore, L. et al. (2008). Early intensive care unit mobility therapy in the treatment of acute respiratory failure. *Crit Care Med, 36*(8), 2238-43.
50. National Institute for Health and Clinical Excellence. (2009). Critical Illness Rehabilitation Guidelines. NICE clinical guideline 83.
51. Oesch, P., Hilfiker, R., Keller, S., Kool, J., Schädler, S., Tal-Akabi, A., et al. (2007). *Assessments in der muskuloskelettalen Rehabilitation* (1. Auflage). Bern: Verlag Hans Huber.
52. Pohlman, MC., Schweickert, WD., Pohlman. AS., Nigos, C., Pawlik, AJ., Esbrook, CL. et al. (2010). Feasibility of physical and occupational therapy beginning from initiation of mechanical ventilation*. Crit Care Med, 38*(11), 2089-94.
53. Radlinger, L., Bachmann, W., Homburg, J., Leuenberger, U. & Thaddey, G. (1998). *Rehabilitatives Krafttraining.* Stuttgart: Georg Thieme Verlag.
54. Redelmeier, DA., Bayoumi, AM., Goldstein, RS., Guyatt, GH. (1997). Interpreting Small Differences in Functional Status: The Six Minute Walk Test In Chronic Lung Disease Patients. *Am J Respir Crit Care Med,. 155*(4), 1278-82.
55. Schädler, S., Kool, J., Lüthi, H., Marks, D., Oesch, P., Pfeffer. A. & Wirz, A. (2006*). Assessments in der Neurorehabilitation.* (1. Auflage). Bern: Verlag Hans Huber.
56. Schweickert, W.D., Pohlmann, M.C., Nigos, C., Pawlik, A.J., Esbrook, C.L., Spears, L. et al. (2009). Early physical and occupational therapy in mechanically ventilated, critically ill patients: a randomised controlled trial. *Lancet, 373*, 1874-1826.
57. Stevens, RD., Marshall, SA., Cornblath, DR., Hoke, A., Needham, DM., de Jonghe, B. et al. (2009). A framework for diagnosing and classifying intensive care unit-acquired weakness. *Crit Care Med, 37*(10), 299-308.
58. Stockley, RC., Morrison, J., Rooney, J. & Hughes, J. (2012). Move it or lose it?: A survey of the aims of treatment when using passive movements in intensive care. *Intensive Crit Care Nurs., 28*(2), 82-87.
59. Truong, AD., Fan, E., Brower, RG. & Needham, DM. (2009). Bench-to-bedside review: Mobilizing patients in the intensive care unit – from pathophysiology to clinical trials. *Crit Care,13*(4), 216.
60. Van der Schaaf, M., Beelen, A., Dongelmans, DA., Vroom, MB. & Nollet, F. (2009). Functional stuatus after intensive care: a challenge for rehabilitation professionals to improve outcome. *J Rehabil Med, 41,* 360–366.

61. Winkelman, C., Johnson, KD., Hejal, R., Gordon, NH., Rowbottom, J., Daly, J. et al. (2012). Examining the positive effects of exercise in intubated adults in ICU: A prospective repeated measures clinical study. *Intensive Crit Care Nurs,* in press.
62. Zeppos, L., Patman, S., Berney, S., Adsett, JA., Bridson, JM. & Paratz, JD. (2007). Physiotherapy intervention in intensive care is safe: an observational study. *Aust J Physiother.*, *53*(4), 279-83.
